# SMAD4 maintains the fluid shear stress set point to protect against arterial-venous malformations

**DOI:** 10.1172/JCI168352

**Published:** 2023-09-15

**Authors:** Kuheli Banerjee, Yanzhu Lin, Johannes Gahn, Julio Cordero, Purnima Gupta, Islam Mohamed, Mariona Graupera, Gergana Dobreva, Martin A. Schwartz, Roxana Ola

**Affiliations:** 1 Experimental Pharmacology Mannheim (EPM) and; 2 Department of Cardiovascular Genomics and Epigenomics, European Center for Angioscience (ECAS), Medical Faculty Mannheim, Heidelberg University, Mannheim, Germany.; 3 German Centre for Cardiovascular Research (DZHK), Mannheim, Germany.; 4 Josep Carreras Leukaemia Research Institute (IJC), Badalona, Spain.; 5 Yale Cardiovascular Research Center, Yale School of Medicine, New Haven, Connecticut, USA.

**Keywords:** Vascular Biology, Molecular biology

## Abstract

Vascular networks form, remodel, and mature under the influence of both fluid shear stress (FSS) and soluble factors. Physiological FSS promotes and maintains vascular stability via synergy with bone morphogenic proteins 9 and 10 (BMP9 and BMP10). Conversely, mutation of the BMP receptors activin-like kinase 1 (*ALK1*), endoglin (*ENG*), or the downstream effector, SMAD family member 4 (*SMAD4*) leads to hereditary hemorrhagic telangiectasia (HHT), characterized by fragile and leaky arterial-venous malformations (AVMs). How endothelial cells (ECs) integrate FSS and BMP signals in vascular development and homeostasis and how mutations give rise to vascular malformations is not well understood. Here, we aimed to elucidate the mechanism of synergy between FSS and SMAD signaling in vascular stability and how disruption of this synergy leads to AVMs. We found that loss of *Smad4* increased the sensitivity of ECs to flow by lowering the FSS set point, with resulting AVMs exhibiting features of excessive flow-mediated morphological responses. Mechanistically, loss of SMAD4 disinhibits flow-mediated KLF4-TIE2-PI3K/Akt signaling, leading to cell cycle progression–mediated loss of arterial identity due to KLF4-mediated repression of cyclin dependent Kinase (CDK) inhibitors *CDKN2A* and *CDKN2B*. Thus, AVMs caused by *Smad4* deletion are characterized by chronic high flow remodeling with excessive EC proliferation and loss of arterial identity as triggering events.

## Graphical abstract

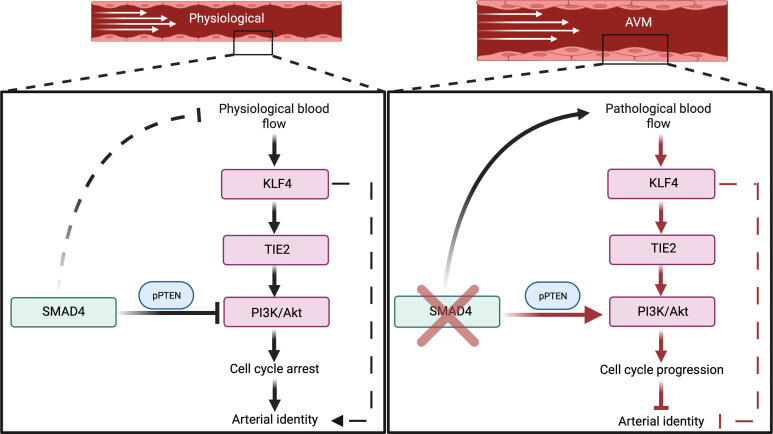

## Introduction

Vascular networks form, remodel, and mature under the influence of multiple biomechanical and biochemical stimuli, but how are they integrated to mediate vascular development and adult homeostasis is poorly understood. Fluid shear stress (FSS) from blood flow is a critical variable that determines vascular endothelial cell (EC) number, shape, and fate in vascular development and maintenance (1, 2). ECs also polarize and migrate according to the flow direction; in different systems this may be with or against the flow (3); in the developing retina, polarization is against the flow, which is proposed to be important in guiding vessel formation (4, 5). One key aspect of EC flow responses is the existence of a cell-autonomous shear stress set point specific to each vessel type. FSS near the set point, i.e., physiological FSS (P-FSS) promotes EC elongation and alignment parallel to the flow and induces cell cycle arrest, thus stabilizing the vessel. Indeed, flow that is persistently above (H-FSS) or below (L-FSS) this level triggers vessel remodeling to restore FSS to the appropriate magnitude (6). VEGFR3 expression levels and noncanonical WNT are among the mechanisms proposed to regulate the FSS set point for EC alignment and axial EC polarity, respectively (6, 7). But how the FSS set point is regulated for other EC responses in different types of vessels and the roles that these mechanisms play in disease are largely unknown.

Shear stress and bone morphogenic proteins (BMP) 9 and BMP10 act in a fine-tuned bidirectional feedback loop to promote EC quiescence and vascular homeostasis. On one hand, P-FSS sustains BMP9-induced activation of canonical SMAD1 and 5 in mature vessels with low levels of circulating BMP9. On the other hand, BMP9 contributes to the inhibition of EC proliferation by P-FSS and to expression of factors that mediate pericyte recruitment (8). Its perturbation via mutation of key intermediates such as activin-like kinase 1 (*Alk1*), endoglin (*Eng*)*,* or Smad family member 4 (*Smad4)* leads to loss of EC quiescence and formation of focal arterial-venous malformations (AVMs).

AVMs are direct connections of feeding arteries to draining veins without an intermediate capillary bed. These lesions are a pathological characteristic of inherited hereditary hemorrhagic telangiectasia (HHT), a vascular disorder caused by heterozygous loss-of-function (LOF) mutations in BMP9 and BMP10 signaling pathway genes including those encoding the ligand, BMP9; the type I BMP receptor, ALK1; the auxiliary coreceptor, ENG; and the transcriptional effector, SMAD4 (9–11). Recently, we, and others, have developed genetic and pharmacological murine models for HHT. These models have uncovered 2 important aspects of these disorders: (a) that AVMs are exclusively a pathogenic feature of canonical BMP9/10 signaling (8, 12–14), and (b) that formation of HHT-like AVMs in the aforementioned models is aggravated by flow-induced shear stress–mediated phosphatidylinositol 3-kinase (PI3K) pathway overactivation (15–17).

Mechanistically, disrupted SMAD4 signaling in ECs leads to increased PI3K/Akt activity through loss of transcriptional repression of casein kinase (CK) 2-mediated phosphatase and tensin homolog (PTEN) phosphorylation (16). Yet, how dysregulation of this pathway leads to AVMs is not well understood. Under normal conditions, PI3K/Akt activation by P-FSS is important for multiple downstream responses, including cell survival and cell alignment, but also inducing activation of NOS3 in flow-mediated vasodilation (18, 19). Conversely, dysregulation of this pathway in AVMs is characterized by alteration in EC size and fate, misdirected migration, and increased proliferation (8, 15–17, 20, 21), events that are potentially due to dysregulated EC flow responses. But causal association between shear stress responses, PI3K/Akt, and AVM formation remains to be established and the mechanisms discovered.

These findings prompted us to investigate whether SMAD4 signaling maintains EC quiescence and vascular stability through regulation of the FSS set point to restrict PI3K/Akt-mediated flow responses.

Here, we report that SMAD4 regulates FSS set point–mediated EC quiescence responses by restricting flow-induced Krüppel-like transcription factor (KLF4). KLF4 overexpression after *Smad4* loss mediates excessive PI3K/Akt pathway activation in ECs through transcriptional regulation of *TEK* tyrosine kinase (TIE2). Further, loss of P-FSS-induced cell cycle arrest in G1 due to excessive KLF4-mediated repression of cyclin dependent kinase (CDK) inhibitors and resultant loss of arterial identity is a key driver of AVM formation upon *Smad4* depletion.

## Results

### Smad4 signaling maintains the shear stress set point–mediated EC responses.

In developing vasculature, AVMs form in regions of high blood flow (8, 16, 22). To gain insights into the contribution of FSS to AVM pathogenesis, we performed RNA-Seq in control (*CTRL* siRNA) and *SMAD4* siRNAs primary human umbilical cord ECs (HUVECs) under static conditions or under P-FSS (12 DYNES/cm^2^) for 24 hours.

**Figure 1:**
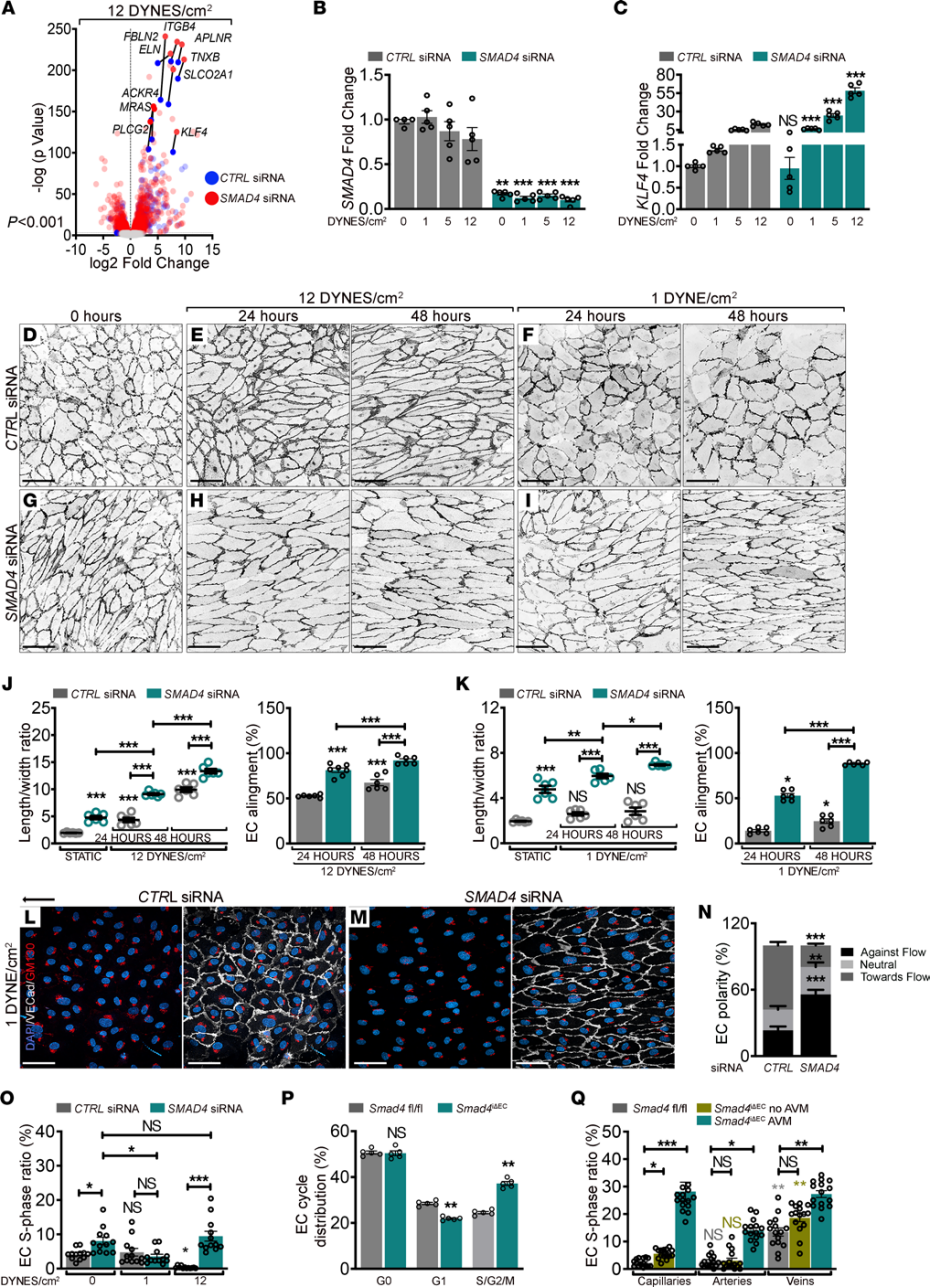
SMAD4 maintains the FSS set point to restrict flow-mediated EC responses. (**A**) Volcano plot highlighting the 10 most significantly upregulated genes upon 24 hours 12 DYNES/cm^2^ FSS in *CTRL* and *SMAD4* siRNA HUVECs (*n* = 3/group). Color key shows log_2_ change after *SMAD4* depletion. (**B** and **C**) qPCR for *SMAD4* (**B**) and *KLF4* (**C**) in *CTRL* and *SMAD4* siRNAs HUVECs grown in static versus 1, 5, and 12 DYNES/cm^2^ FSS for 24 hours (*n* = 5/group). (**D**–**I**) VE-Cadherin staining (negative images) of *CTRL* (**D**–**F**) and *SMAD4* (**G**–**I**) siRNAs HUVECs grown in static (**D** and **G**), subject to 12 DYNES/cm^2^ (**E** and **H**) or 1 DYNE/cm^2^ (**F** and **I**) for 24 and 48 hours. Flow direction: right to left. (**J** and **K**) Quantification of length-to-width ratio and EC alignment parallel to flow direction (%) in *CTRL* and *SMAD4* siRNA HUVECs in static and 12 DYNES/cm^2^ (**J**) or 1 DYNE/cm^2^ (**K**) (*n* = 6 average of 3 images (70–140 cells/image) per 3 independent experiments/group). (**L** and **M**) VE-Cadherin (white), GM130 (red), and DAPI (blue) staining of *CTRL* and *SMAD4* siRNA HUVECs upon 24 hours 1 DYNE/cm^2^. Arrow indicates flow direction. (**N**) Quantification of EC polarization (%) against, with the flow’s direction, or nonoriented (neutral) in experiments from **L** and **M** (*n* = 12 images [50–100 cells/image] per 3 independent experiments/group). (**O**) S-phase ratio (EdU^+^) per total number of DAPI^+^ nuclei (%) in *CTRL* and *SMAD4* siRNA HUVECs grown in static, 1, and 12 DYNES/cm^2^ for 24 hours (*n* = 12 images [200–250 cells/image] per 3 independent experiments/group). (**P**) FACS analysis in *Smad4^fl/fl^ and* *Smad4*^iΔEC^ P6 retinas (*n* = 5 retinas/group). (**Q**) S-phase ratio (EdU^+^/ERG^+^) per total ECs (ERG^+^) in capillaries, arteries, and veins of *fl/fl* and *Smad4*^iΔEC^ retinas engaged or not in AVMs (*n* = 15 images from 4 retinas/group). Scale Bars: 100μm in panels **D**–**I**, **L**, and **M**. Data are represented as mean ± SEM. Mann-Whitney test (**B**, **C**, **N**, and **P**) and 1-way Anova (**J**, **K**, **O**, and **Q**) were used to determine statistical significance. *P < 0.05,**P < 0.01,***P < 0.001.

Interestingly, *KLF4* was the top transcription factor and among the 10 most significantly FSS-induced genes upon *SMAD4* depletion (Figure 1A). These genes were validated by quantitative PCR (qPCR) (Supplemental Figure 1A; supplemental material available online with this article; https://doi.org/10.1172/JCI168352DS1). As KLF4 is well known to be induced by FSS and to regulate the expression of many shear-responsive genes (23, 24), it may mediate AVM pathogenesis. We therefore assessed *KLF4* induction in HUVECs subjected to increasing magnitudes of laminar FSS (1, 5, and 12 DYNES/cm^2^) ± *SMAD4* depletion (confirmed in Figure 1B). Notably, *SMAD4* loss augmented the dose-dependent flow induction of *KLF4* (Figure 1C). These data suggest that SMAD4 restricts flow-induced activation of KLF4 to perhaps restrain flow-mediated EC quiescence responses.

To explore this hypothesis, we examined HUVECs transfected with *SMAD4* or *CTRL* siRNA under P-FSS (12 DYNES/cm^2^) and L-FSS (1 DYNE/cm^2^) for 24 and 48 hours (Figure 1, D–I). *SMAD4*-depleted HUVECs were already elongated (nondirectionally) without flow (0 hours), as assessed by the length-to-width ratio of individual ECs (Figure 1, D and G); cells under 12 DYNES/cm^2^ for 24 hours elongated further and aligned better parallel to the direction of flow (≤ 30° angle–directionality**)** compared with *CTRL* ECs (Figure 1, E and H; quantified in Figure 1J). Remarkably, *SMAD4*-depleted HUVECs under 1 DYNE/cm^2^ elongated and aligned, whereas no significant changes were observed in *CTRL* HUVECs even at 48 hours (Figure 1, F and I; quantified in Figure 1K). Thus, *SMAD4* knock down (KD) potently augments flow-induced EC elongation and alignment.

ECs show flow magnitude–dependent polarization and migration against the flow direction (defined as axial polarity) (4). To identify the function of SMAD4 in FSS-mediated polarization, HUVECs depleted for *SMAD4* versus *CTRL* were subject to 1 DYNE/cm^2^ FSS and labeled for GM130 (to detect Golgi apparatus), VE-Cadherin (cell junctions) and DAPI (nuclei). Interestingly, measuring the nucleus-Golgi axis angle (in an angle ≤ 45° in relation to flow direction–EC orientation), *SMAD4-*KD HUVECs polarized more against the flow than *CTRL* cells (Figure 1, L and M; quantified in Figure 1N). Thus, *SMAD4* depletion amplified EC elongation, alignment, and orientation in response to FSS, indicating that SMAD4 restricts flow-induced morphological responses.

P-FSS also inhibits EC proliferation by inducing cell cycle arrest in G1 to maintain vessel stability (25). To test the role of SMAD4 in this process, we performed EdU labeling to identify ECs in S phase in static, L-FSS, and P-FSS. *SMAD4* depletion slightly increased the baseline cell cycle progression under static conditions. Under 1 DYNE/cm^2^, *SMAD4* depletion moderately decreased cell cycle progression relative to static *SMAD4-*KD cells. Under 12 DYNES/cm^2^, *SMAD4* KD converted the inhibition of proliferation into stimulation (Supplemental Figure 2A; quantified in Figure 1O). As presented in the Discussion, these results can be interpreted in terms of changes in the FSS set point.

To validate these results in vivo, we analyzed retinal EC cycle distribution by FACS from *Smad4^fl/fl^* and EC-specific Tamoxifen-inducible *Smad4* depleted postnatal day 6 (P6) neonates (*Smad4*^iΔEC^ or *Smad4*EC KO). *Smad4*^iΔEC^ retinas revealed an increase in ECs in S/G2/M with a concomitant decrease in G1 (Figure 1P). As retinal AVMs form with an increased prevalence close to the optic nerve, we further quantified the total number of ECs per mm^2^ and the proportion of ECs in S-phase (EdU^+^ERG^+^/ERG^+^) and M-phase (PH3^+^ERG^+^/ERG^+^) in *Smad4^fl/fl^* and *Smad4*^iΔEC^ vascular plexus. Whereas in vascular plexus outside AVMs, total EC number was decreased with a slight increase in S/G2/M, within AVMs, total ECs were higher with higher fraction of cells in S-phase (Supplemental Figure 2B; quantified in Supplemental Figure 2, C and D) and M-phase (Supplemental Figure 2E; quantified in Supplemental Figure 2F). Focusing on different vascular beds within the plexus, we found that in *Smad4^fl/fl^*, only venous ECs were EdU positive. In *Smad4*EC-KO retinas, in regions other than AVM, venous capillaries and capillary ECs showed a slight increase in EdU labeling while the arterial ECs remained arrested. However, within the AVMs, ECs were excessively proliferating (Supplemental Figure 2B; quantified in Figure 1Q). Together, these results suggest that *Smad4*-depleted ECs outside AVMs are arrested in S/G/M phase and actively proliferating only in the AVM regions, likely due to loss of flow-mediated cell cycle arrest.

### KLF4 mediates flow-induced hyper-responsiveness of SMAD4-depleted HUVECs.

As *SMAD4* depletion augmented KLF4 expression under flow, we next considered its role in altered flow-mediated responses.

**Figure 2:**
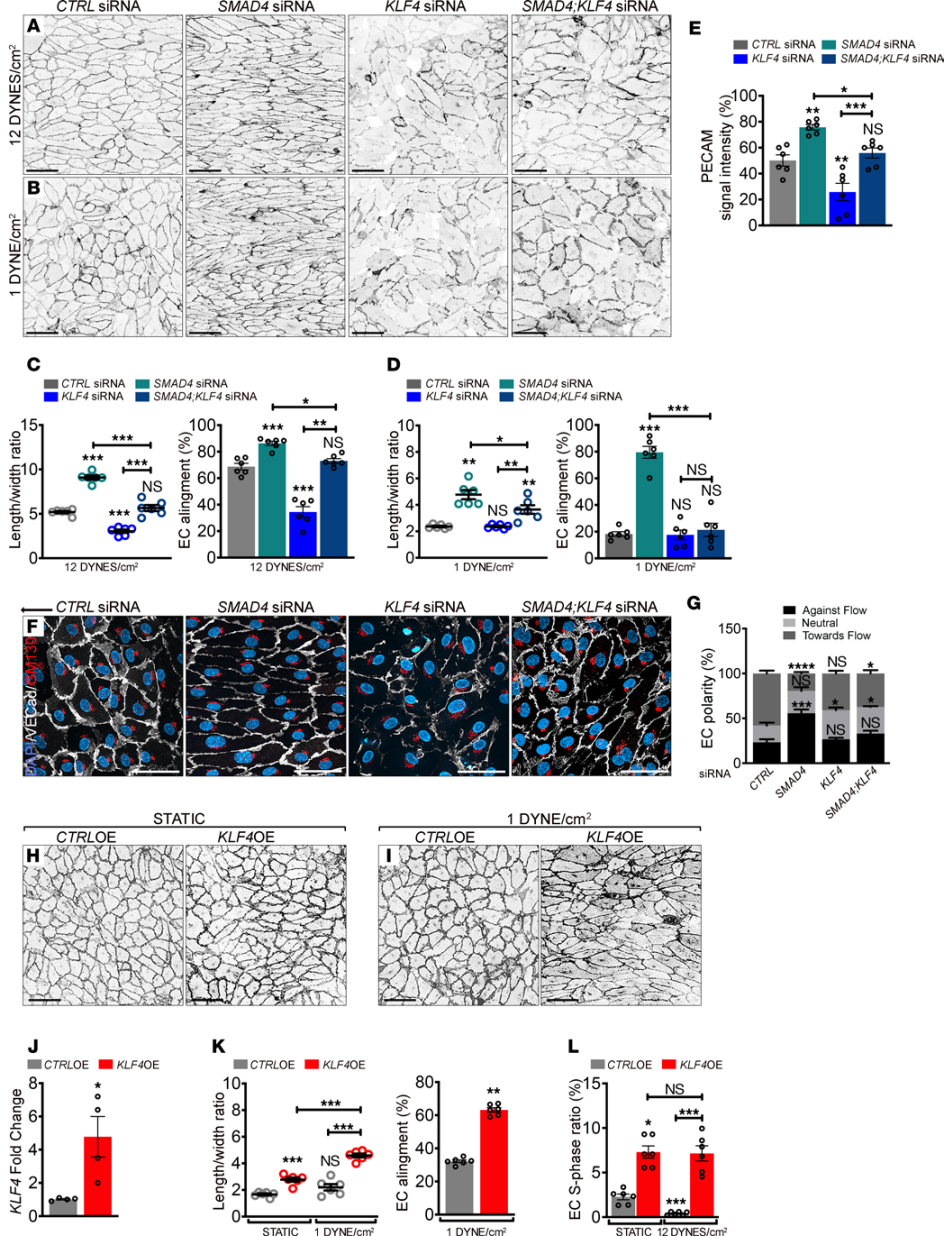
KLF4 mediates the flow-induced hyperresponsiveness of *SMAD4*-depleted cells. (**A** and **B**) Representative PECAM staining (negative images) of *CTRL*, *SMAD4,* and *KLF4* and *SMAD4;KLF4* siRNAs HUVECs subject to 12 DYNES/cm^2^ (**A**) and 1 DYNE/cm^2^ (**B**) for 24 hours. (**C** and **D**) Quantification of the length-to-width ratio and of EC alignment parallel to the flow direction (%) from experiments in **A** and **B** (average of *n* = 6 images (70–140 cells/image) per 3 independent experiments/group). (**E**) Quantification of PECAM signal intensity from experiments in **A** (*n* = 6 images per 3 independent experiments/group). (**F**) Colabeling for PECAM (white), GM130 (red), and DAPI (blue) of *SMAD4* and *KLF4* and *SMAD4;KLF4* siRNAs HUVECs subject to 24 hours 1 DYNE/cm^2^. Black arrow indicates flow direction from right to left. (**G**) Quantification of cell polarization: against flow direction, with flow, or neutral (nonoriented) in pictures, as shown in **F** (*n* = 6 images (50–100 cells/image) per 3 independent experiments/group). (**H** and **I**) Representative VE-Cadherin staining (negative images) of HUVECs transfected with an empty lentiviral construct (*CTRL* OE) and an overexpression lentivirus for *KLF4* (*KLF4* OE) grown in static (**H**) or subject to 1 DYNE/cm^2^ for 48 hours (**I**). (**J**) *KLF4* expression by qPCR (fold change) in *CTRL-*OE and *KLF4-*OE HUVECs (*n* = 4/group). (**K**) Quantification of the length-to-width ratio and of EC alignment parallel to the flow direction (%) in images as shown in **H** and **I** (*n* = 6 images (70–140 cells/image) per 3 independent experiments/group). (**L**) S-phase ratio ((EdU^+^) per total number of DAPI^+^ nuclei (%)) of *CTRL-*OE and *KLF4-*OE HUVECs grown in static and 12 DYNES/cm^2^ for 24 hours (*n* = 6 images (140–250 cells/image) per 3 independent experiments/group). Scale Bars: 100μm in **A**, **B**, **H**, and **I**, and 50μm in **F**. Data are represented as mean ± SEM. Mann-Whitney test (**J** and **K**-right), 1-way Anova (**C**, **D**, **E**, **K**-left, **L**), and Kruskal-Wallis tests (**G**) were used to determine statistical significance.*P < 0.05, **P < 0.01, ***P < 0.001.

HUVECs were depleted for *SMAD4,* *KLF4,* or both, and 48 hours after transfection, cells were subjected to 1 or 12 DYNES/cm^2^ for 24 hours. *KLF4-*KD cells failed to elongate and align under all conditions (Figure 2, A and B; quantified in Figure 2, C and D), but increased in cell size (Figure 2, A and B; quantified in Supplemental Figure 3, A and B) with loss of PECAM (Figure 2, A and B; quantified in Figure 2E) but not VE-Cadherin (Supplemental Figure 3C; quantified in Supplemental Figure 3D) at cell-cell junctions. *KLF4* depletion reversed the excessive elongated morphology and cell directionality of *SMAD4-*depleted HUVECs (Figure 2, A and B; quantified in Figure 2, C and D) but further increased the total cell area of elongated *SMAD4-*KD cells (Figure 2, A and B; quantified in Supplemental Figure 3, A and B). Interestingly, loss of *KLF4* normalized PECAM expression in *SMAD4-*KD cells (Figure 2, A and B; quantified in Figure 2E). *KLF4* depletion also rescued the excessive orientation of *SMAD4-*KD cells under 1 DYNE/cm^2^ flow (Figure 2F; quantified in Figure 2G).

To determine if these morphological effects occur in a cell autonomous manner, we overexpressed *KLF4* using a lentiviral construct (*KLF4* OE; confirmed in Figure 2J) and measured flow-mediated events. *KLF4* OE increased EC elongation without flow, which was further enhanced under 1 DYNE/cm^2^ for 24 hours (Figure 2, H and I; quantified in 2K). *KLF4-*OE cells also aligned better in the flow direction (Figure 2I; quantified in 2K). Measurement of EdU incorporation showed that *KLF4* OE increased cell cycle progression at baseline and under P-FSS (Figure 2L). *KLF4* OE thus phenocopies key aspects of *SMAD4* KD. Together, these results show that hyper-induction of KLF4 contributes to effects of *SMAD4* LOF.

### KLF4 is a key determinant in AVM formation.

**Figure 3:**
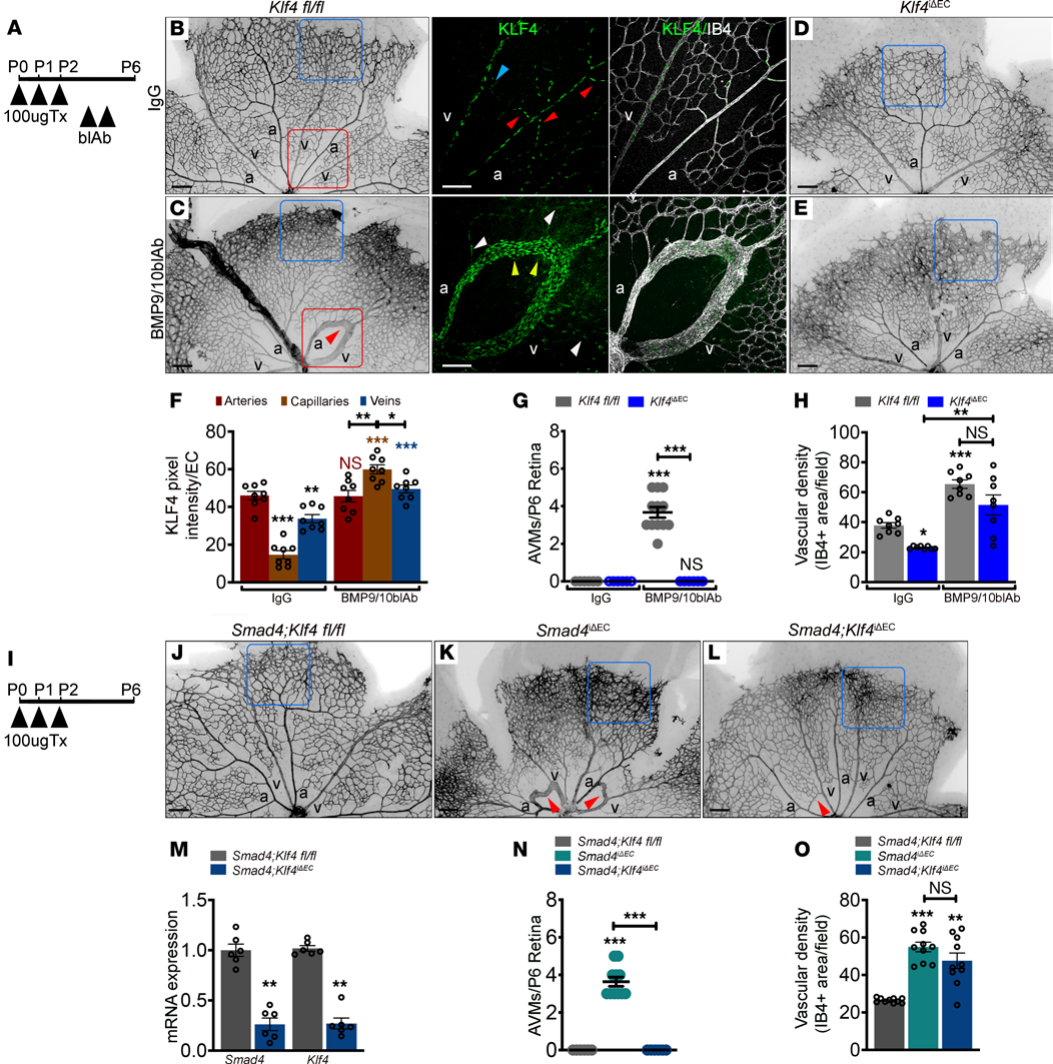
Flow-induced KLF4 is a key determinant in AVM formation. (**A**) Schematic of the experimental strategy. Arrowheads indicate intragastric injection of 100μg tamoxifen (Tx) at P0–P2 in *Klf4^fl/fl^* and *Klf4*^iΔEC^, i.p. injection of IgG and blocking antibodies for BMP9 and BMP10 (BMP9/10blAb) at P4 and P5 and analysis at P6. (**B**–**E**) Representative IB4 labeling (negative images) of P6 *Klf4^fl/fl^* (**B** and **C**) and *Klf4*^iΔEC^ (**D** and **E**) retinas treated with IgG (**B** and **D**) and BMP9/10blAb (**C** and **E**). Higher magnification of the small insets (red squares) from **B** and **C** labelled for KLF4 (green) and IB4 (white). Small red/blue arrowheads in **B** indicate the branch points in arteries and veins. Yellow/white arrowheads in **C** indicate increased KLF4 intensity in AVM capillary ECs and lower KLF4 in vessels outside of AVMs, respectively. (**F**) Quantification of KLF4 pixel intensity per EC in arteries, capillaries, and veins in *Klf4^fl/fl^* retinas treated with IgG and BMP9/10blAb (*n* = 8 images from 4 retinas/group). (**G** and **H**) Quantification of P6 AVM numbers (**G**) (*n* = 6 retinas/group) and of vascular density at the retinal front (%) (**H**) (*n* = 8 retinas/group) in the indicated genotypes. (**I**) Schematic of the experimental strategy used to delete *Smad4* and *Klf4* in neonates (P0–P6). (**J–L**) IB4 labeling (negative images) of P6 *Smad4;Klf4^fl/fl^* (**J**), *Smad4*^iΔEC^ (**K**), and *Smad4;Klf4*^iΔEC^ (**L**) retinas. (**M**) *Smad4* and *Klf4* mRNA expression in mouse lung ECs (mLECs) from P6 mice (*n* = 6 /group). (**N** and **O**) Quantification of AVM numbers (**N**) (*n* = 6 retinas/group) and of vascular density at the retinal front (%) (**O**) (*n* = 10 (2 images per retina)/group) of the indicated genotypes. Red arrows in **C**, **K**, and **L** mark AVMs. Blue squares in **B**–**E** and **J**–**L** indicate the vascular front. Scale Bars: 500 μm in **B**–**E** and **J**–**L** and 100 μm in higher magnification images from **B** and **C**. a, artery; v, vein. Data are represented as mean ± SEM. Statistical significance was determined by Mann-Whitney test (**M**) and 1-way Anova (**F**, **G**, **H**, **N**, and **O**).*P < 0.05, **P < 0.01, ***P < 0.001.

To test KLF4 in AVM pathogenesis, we used 2 in vivo models of AVM formation in which we compared EC-specific Tx-inducible *Klf4* LOF (*Klf4*^iΔEC^) to *Klf4^fl/fl^* neonatal retinas. AVMs were induced by administration of blocking antibodies for BMP9 and BMP10 (blAb BMP9/10) with nonspecific IgG as control (Figure 3A). Blockade of BMP9/10 in *Klf4^fl/fl^* retinas induced AVMs (average of 3.6–4 AVMs per retina) and increased vascular density at the front (IB4 positive area per field) (Figure 3, B and C; quantified in Figure 3, G and H). EC KO of *Klf4* moderately reduced vascular density in IgG-treated controls and had no effect on increased density after BMP9/10 blockade (Figure 3, B–E; quantified in Figure 3H), however, *Klf4*EC KO completely rescued AVMs in pups treated with BMP9/10 blAbs (Figure 3, C and E; quantified in Figure 3G).

Labeling *Klf4^fl/fl^ and Klf4*^iΔEC^ retinas for KLF4 confirmed antibody specificity (Supplemental Figure 4A). In *Klf4^fl/fl^*-treated retinas, KLF4 was upregulated by FSS in high-flow regions, with the highest induction at the first arterial branch points and arteries, lower expression in veins and venous branch points, and very little in capillaries in *Klf4^fl/fl^* (red/blue arrowheads in small inset, Figure 3B; quantified in Figure 3F). In contrast to IgG-treated, in *Klf4^fl/fl^* retinas treated with blAb BMP9/10, KLF4 was highly upregulated in AVM capillary ECs relative to the feeding artery and vein (yellow arrowheads in small inset Figure 3C; quantified in Figure 3F). Vessels not engaged in AVMs (white arrowheads in small inset Figure 3C) showed lower KLF4 intensity, suggesting, on one hand, lower flow outside of AVMs, as previously reported (26), and on the other hand, that FSS-induced *Klf4* expression is partially restrained by SMAD4 signaling, supporting our RNA-Seq data. Together, these results emphasize that high KLF4 within AVM ECs is likely a consequence of increased sensitivity of ECs to flow.

To validate AVM rescue upon *Klf4* inactivation in a genetic model of *Smad4* deletion, we created EC-specific Tx-inducible double-KO mice, *Smad4;Klf4*^iΔEC^. Tx was injected at P0–P2 and retinas were analyzed at P6 (Figure 3I). Efficient *Smad4* and *Klf4* gene deletion was validated by qPCR from P6 mouse lung endothelial cells (mLECs; Figure 3M). *Klf4* inactivation also rescued AVM formation in *Smad4*^iΔEC^ retinas but not the excessive sprouting at the vascular front (Figure 3, J–L; quantified in Figure 3, N and O).

Thus, excessive flow-induced KLF4 is a key determinant in AVM pathogenesis upon inactivation of either the ligands or the transcriptional effector.

### KLF4 mediates the shear stress–induced aberrant EC events in vivo.

**Figure 4:**
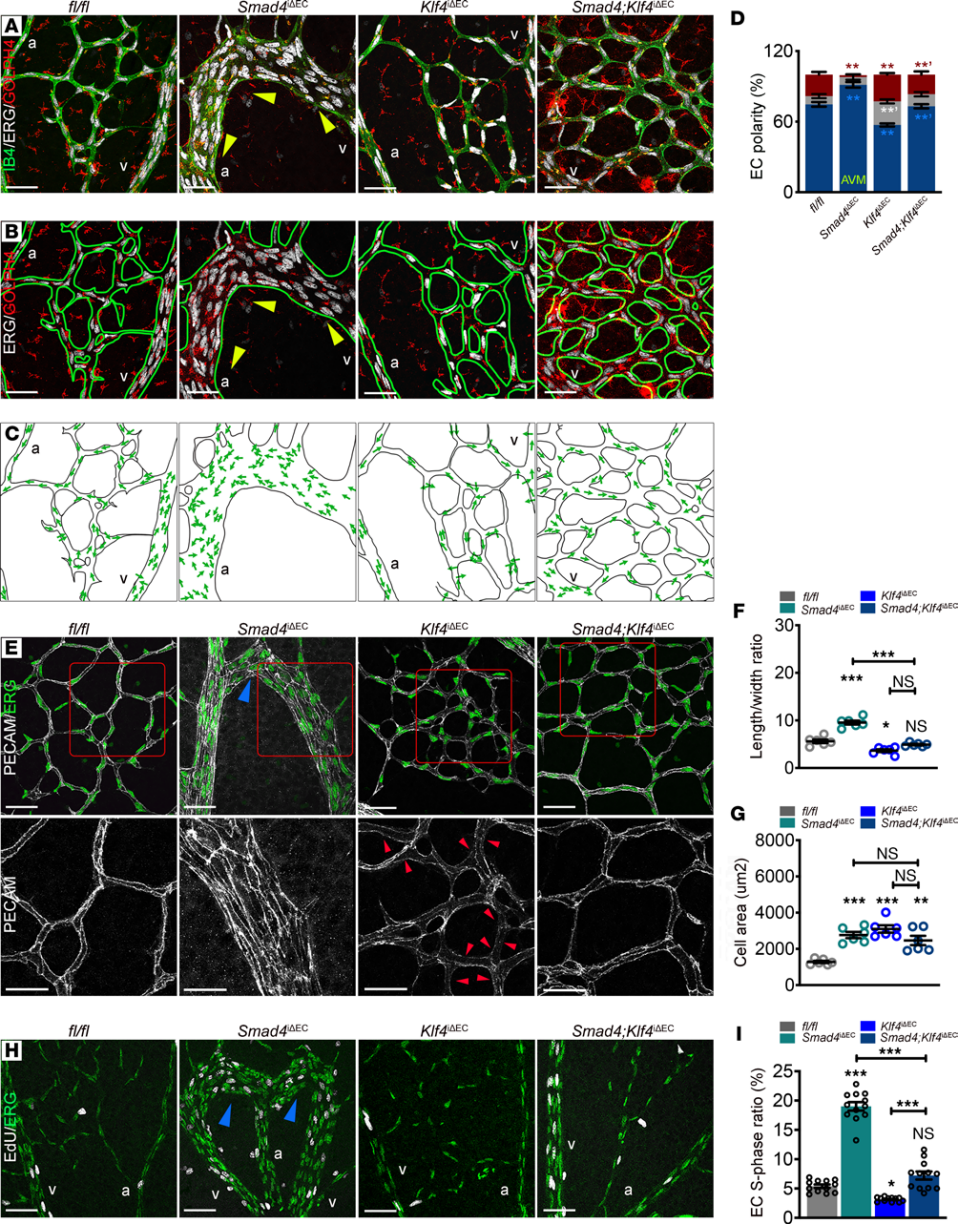
KLF4 mediates the shear stress–induced aberrant EC events within AVMs. (**A**–**C**) Representative confocal images of P6 Tx induced *fl/fl*, *Smad4*^iΔEC^, *Klf4*^iΔEC^, *and Smad4;Klf4*^iΔEC^ retinal plexus labeled for ERG (nuclei-white), GOLPH4 (golgi-red), and IB4 (green) (**A**) and ERG, GOLPH4, and IB4 (green line) (**B**). Yellow arrowheads indicate the orientation of ECs within the AVM against the flow direction from vein toward the artery. (**C**) Panels illustrating EC polarization based on position of golgi in relation to the nucleus in the direction of migration (green arrows). (**D**) Quantification of EC polarization: against, with flow, and neutral (nonpolarized) in capillaries from P6 Tx-induced retinas from the indicated genotypes (*n* = 3 retinas/group). (**E**) Upper panel: confocal images of P6 Tx-induced *fl/fl*, *Smad4*^iΔEC^, *Klf4*^iΔEC^, and *Smad4;Klf4*^iΔEC^ retinal plexus labeled for PECAM (white) and ERG (green). Lower panel: magnified pictures (red insets in upper panels) labeled for PECAM (white). Small red arrowheads indicate loss of PECAM at cell-cell junctions in *Klf4*^iΔEC^ retinas. (**F** and **G**) Quantification of length/width ratio (**F**) and of cell area (**G**) in capillary ECs in the indicated genotypes (*n* = 6 [2 images (average of 50–100 cells/image) per retina]/group). (**H**) Representative labeling for EdU (white) and ERG (green) in vascular plexus of retinas from P6 *fl/fl*, *Smad4*^iΔEC^*, Klf4*^iΔEC^, and *Smad4;Klf4*^iΔEC^ mice. Blue arrowheads in **E** and **H** indicate AVMs. (**I**) S-phase ratio (EdU^+^/ERG^+^) per total amount of ECs (ERG^+^) in the vascular plexus of the indicated genotypes (%) (*n* = 12 images from 4 retinas/group). Scale Bars: 50μm in **A**–**C**, **E** (upper panel) and **H**; 20μm in magnified images (lower panel) from **E**. 1-way Anova (**D**, **F**, **G**, and **I**) was used to determine statistical significance. Data are represented as mean ± SEM. *P < 0.05, **P < 0.01, ***P < 0.001. a, artery; v, vein.

To analyze the role of KLF4 in flow-mediated EC events in vivo, we examined axial polarity in *fl/fl*, *Smad4*^iΔEC^*, Klf4*^iΔEC^, and *Smad4*;*Klf4*^iΔEC^ retinas by measuring Golgi orientation. Compared with *fl/fl* mice, *Klf4*-deficient capillaries showed reduced polarization against the flow direction; in *Smad4*^iΔEC^ retinas, *Klf4* inactivation blunted the increased axial polarity (Figure 4, A–C; quantified in Figure 4D). *Klf4* LOF led to loss of PECAM at cell junctions (red arrowheads in Figure 4E) and a decrease in capillary EC elongation with an increased in EC area. However, despite the diminished elongation and orientation of *Smad4*^iΔEC^ capillary ECs by *Klf4* inactivation, cell area in *Smad4*EC-KO capillaries was unaffected (Figure 4E; quantified in Figure 4, F and G), thus confirming our in vitro findings.

We next assayed KLF4-mediated EC cycle progression by EdU labeling of *fl/fl*, S*mad4*^iΔEC^, *Klf4*^iΔEC^, and S*mad4*;*Klf4*^iΔEC^ P6 pups (Figure 4H). Interestingly, *Klf4* inactivation slightly but significantly decreased EdU^+^/ERG^+^ cells and reduced the excessive EdU labelling in the vascular plexus of *Smad4*^iΔEC^ retinas to levels comparable to *fl/fl* retinas (Figure 4H; quantified in Figure 4I). Elevated KLF4 thus contributes to morphological responses and excessive cell cycle progression both in vitro and in *Smad4*EC-KO AVMs.

### Flow-induced KLF4 acts upstream of the mechanosensory complex–PI3K/Akt pathway.

**Figure 5:**
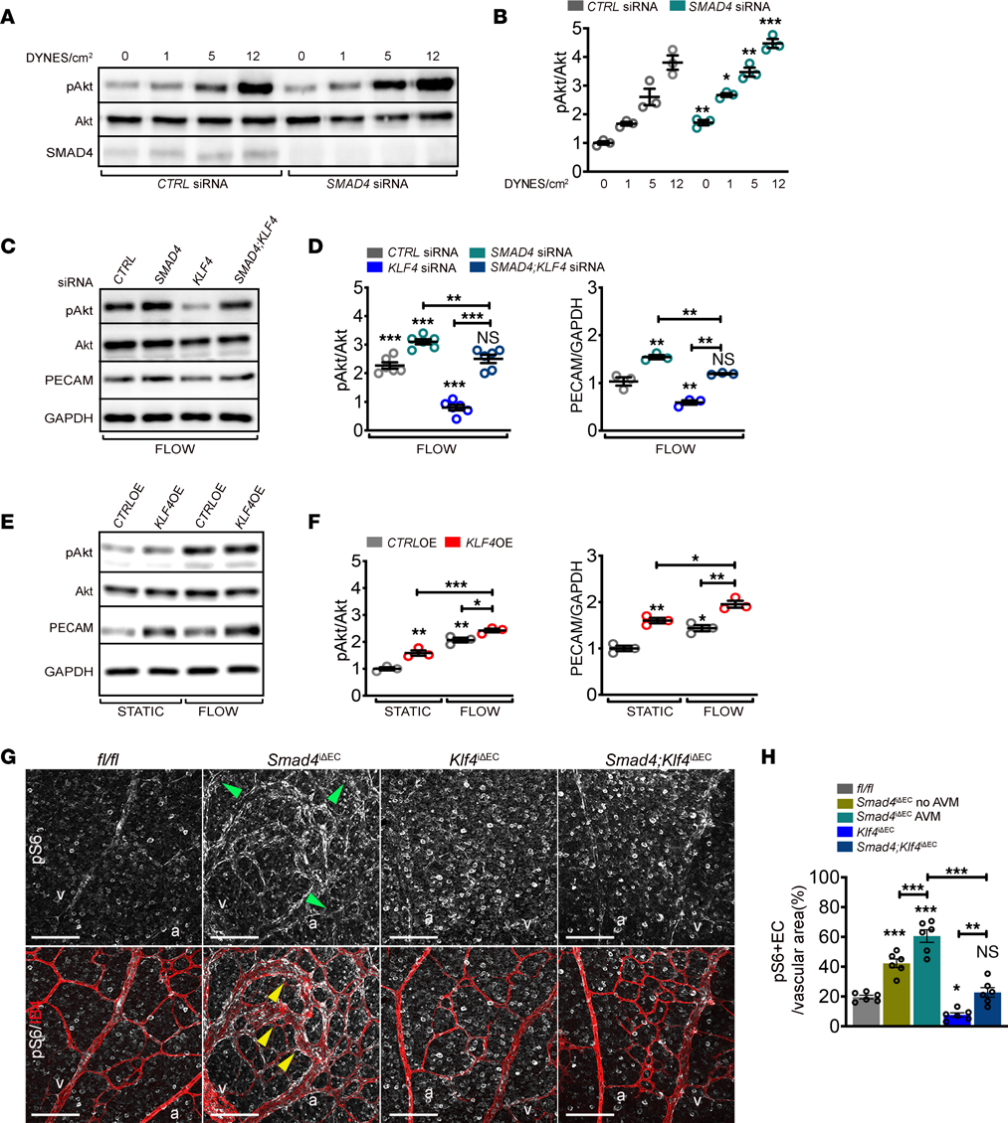
Flow-induced KLF4 acts upstream of mechanosensory complex PI3K/Akt. (**A**) Western Blot (WB) for pAkt, Akt, and SMAD4 of HUVECs transfected with *CTRL* or *SMAD4* siRNAs grown in static or subject to 1, 5, and 12 DYNES/cm^2^ for 4 hours. (**B**) Quantifications of pAkt levels normalized to total Akt for the indicated conditions (*n* = 3/group). (**C**) WB for pAkt, total Akt, PECAM, and GAPDH of *CTRL*, *SMAD4, KLF4,* and *SMAD4;KLF4* siRNAs HUVECs subjected to 12 DYNES/cm^2^ for 4 hours. (**D**) Quantification of pAkt/Akt (*n* = 6/group) and PECAM/GAPDH (*n* = 3/group) in the indicated genotypes. (**E**) WB for pAkt, total Akt, PECAM, and GAPDH in *CTRL-*OE and *KLF4-*OE cells grown in static versus 12 DYNES/cm^2^ for 4 hours. (**F**) Quantification of pAkt/Akt and PECAM/GAPDH in the indicated genotypes (*n* = 3/group). (**G**) Anti-phosphoS6 (pS6) (white-upper panel) and colabeling for pS6 (white) and IB4 (red) (lower panel) of retinal flat mounts from Tx-induced P6 *fl/fl*, *Smad4*^iΔEC^, *Klf4*^iΔEC^, and *Smad4;Klf4*^iΔEC^. Green and yellow arrowheads indicate non-AVM regions and AVM regions, respectively. (**H**) Quantification of pS6 levels in the vascular plexus of *fl/fl*, *Smad4*^iΔEC^ (in regions free of AVMs and within the AVMs), *Klf4*^iΔEC^, and *Smad4;Klf4*^iΔEC^ (*n* = 6 [2 images/retina]/group). a, artery; v, vein. Scale bars: 50μm in **G**. Mann-Whitney test (**B**), 1-way Anova (**D**, **F**, and **H**) were used to determine statistical significance. Data are represented as mean ± SEM. *P < 0.05, **P < 0.01, ***P < 0.001.

High PI3K/Akt activation upon *Smad4* LOF, further augmented by high FSS, is proposed to drive AVM formation (15, 16). To test the role of PI3K/Akt in increased responsiveness of *SMAD4-*KD cells to flow, we subjected *CTRL* versus *SMAD4-*KD HUVECs to FSS at 1, 5, and 12 DYNES/cm^2^. *SMAD4* deletion increased baseline Akt phosphorylation, which was further elevated with increasing flow (Figure 5A; quantified in Figure 5B).

As PI3K inhibition reduces AVMs in HHT mouse models, we evaluated its requirement in flow-mediated EC responses. Cells were treated with the specific PI3K inhibitor Pictilisib for 48 hours (confirmed in Supplemental Figure 5A) or transfected with *AKT1* siRNA and subject to P-FSS. Inhibition of PI3K/Akt by either method significantly rescued the elongation and alignment in *SMAD4*-depleted HUVECs (Supplemental Figure 5B; quantified in Supplemental Figure 5C).

In vivo, Pictilisib treatment blunted the axial polarity in *Smad4^fl/fl^* and normalized EC orientation in *Smad4*^iΔEC^ retinas (Supplemental Figure 5D; quantified in Supplemental Figure 5E). Pictilisib treatment also rescued the excessive EC EdU labeling in *Smad4*^iΔEC^ vascular plexus (quantified in Supplemental Figure 5F) and in *SMAD4-*KD HUVECs under high flow (quantified in Supplemental Figure 5G). EC SMAD4 thus restrains flow-induced PI3K/Akt and downstream responses including EC elongation and directionality, axial polarity, and cell cycle progression.

As SMAD4 restrains flow activation of both KLF4 and Akt in a similar manner and inactivation of either rescues AVM formation, we next considered their relationship in the context of *SMAD4* depletion and flow. We first tested whether KLF4 was downstream of Akt activation by subjecting HUVECs to flow in the presence of Pictilisib. Real-time PCR (RT-PCR) results showed no effect of Pictilisib on flow-induced *KLF4* (Supplemental Figure 5H). Consistent with this finding, KD of each of the components of the junctional mechanosensory receptor complex (*CD31*, encoding PECAM; *KDR*, encoding VEGFR2*; CDH5*, encoding VE-Cadherin) that mediates flow activation of PI3K (27, 28), also had no effect on *KLF4* induction by flow (Supplemental Figure 5I). Thus, flow-induced *KLF4* expression is not downstream of the mechanosensory junctional receptor complex or PI3K.

We next tested whether KLF4 was upstream of mechanosensory receptor complex and Akt activation. We found that *KLF4* inactivation blunted flow-induced Akt activation and PECAM upregulation and rescued Akt hyperactivation and normalized PECAM in *SMAD4*-depleted HUVECs under flow (Figure 5C; quantified in Figure 5D). Conversely, in *KLF4-*OE HUVECs, KLF4 activated Akt and upregulated PECAM with or without FSS (Figure 5E; quantified in Figure 5F). As PECAM was downregulated in *Klf4*EC-KO capillaries (Figure 4E), to confirm decreased Akt activation in ECs in vivo*,* we labeled retinas for phosphorylated S6 ribosomal protein (pS6), a downstream target of Akt, together with IB4. In *Smad4*^iΔEC^ retinas, ECs involved in AVMs showed higher pS6 staining (indicated by yellow arrows) compared with non-AVM vessels (indicated by green arrows), supporting activation that is further augmented by flow within AVMs. *Klf4* depletion decreased pS6 in ECs and largely rescued pS6 activation in *Smad4*^iΔEC^ mutants (Figure 5G; quantified in Figure 5H). KLF4 is, thus, upstream of the junctional mechanosensory receptor complex and PI3K/Akt to control flow-mediated EC events after *Smad4*EC-KO.

### Flow-induced KLF4 requires TIE2 to activate Akt signalling.

**Figure 6:**
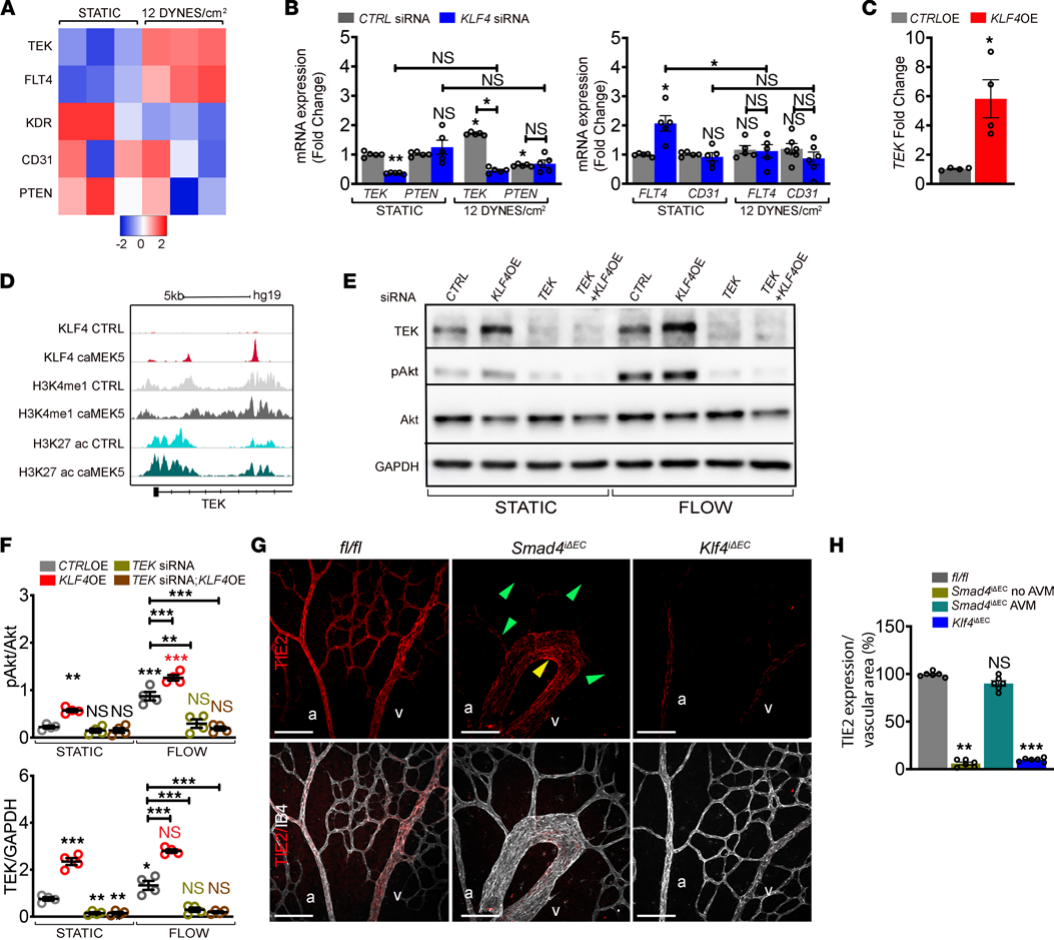
KLF4-mediated TEK expression is required for flow-induced PI3K/Akt activation. (**A**) Heatmap of potential mediators of PI3K signaling in *CTRL* siRNA HUVECs grown in static versus subject to 12 DYNES/cm^2^; *n* = 3/group. Color key shows log_2_ change upon FSS stimulation. (**B**) qPCR for *TEK* and *PTEN* (left panel) and *FLT4* and *CD31* (right panel) in *CTRL* versus *KLF4* siRNAs HUVECs grown in static or subject to 12 DYNES/cm^2^ (*n* = 5/group). (**C**) *TEK* mRNA expression in *CTRL-*OE versus *KLF4-*OE HUVECs (*n* = 4/group). (**D**) Reanalysis of previous published CHIP-Seq data of KLF4 overexpression (caMEK5) in primary human pulmonary artery endothelial cells (PAEC) with the Integrative Genomics Viewer (IGV). Two distinct peaks within enhancer regions of the *TEK* gene were identified. (**E**) WB for indicated proteins of HUVECs transfected with *CTRL* and *TEK* siRNAs and *CTRL-*OE and *KLF4-*OE constructs grown in static or subject to 12 DYNES/cm^2^ for 4 hours. (**F**) Quantification of pAkt/Akt and TEK/GAPDH in indicated genotypes (*n* = 4/group). (**G**) Labeling of Tx-induced P6 *fl/fl*, *Smad4*^iΔEC^ and *Klf4*^iΔEC^ retinas with anti-TIE2 antibody (red) and IB4 (white). Green and yellow arrowheads indicate non-AVM versus AVM region, respectively. (**H**) Quantification of TIE2 expression per vascular area in the indicated genotypes (*n* = 6 [2 images/retina]/group). a, artery; v, vein. Scale Bars: 100μm in **G**. Mann-Whitney test (**C**) and 1-way Anova (**B**, **F**, and **H**) were used to determine statistical significance.Data are represented as mean ± SEM. *P < 0.05, **P < 0.01, ***P < 0.001.

We next addressed mechanisms by which KLF4 regulates PI3K/Akt in the context of flow. As KLF4 is a transcription factor, we reasoned that KLF4 controls expression of an upstream regulator of PI3K activity. Examination of our RNA-Seq data from static *CTRL* and 12 DYNES/cm^2^ conditions for potential modulators of PI3K/Akt identified vascular receptor tyrosine kinases, including *TEK* (coding for TIE2), *FLT4* 9encoding VEGFR3), *KDR,* and *CD31*, all known to contribute to PI3K activation. We also saw changes in PTEN that counteracts PI3K signaling (29) (Figure 6A). While *FLT4* and *TEK* showed upregulation upon flow, *KDR* and *PTEN* were downregulated, and *CD31* mRNA showed no change. To validate these data and regulation upon *KLF4*, we measured their expression in HUVECs depleted for *KLF4* under static versus 12 DYNES/cm^2^. RT-PCR confirmed elevated *TEK* and decreased *PTEN* induction upon flow in *CTRL* cells, while *FLT4* and *CD31* remained unchanged (Figure 6B). In *KLF4-*KD cells, flow failed to upregulate *TEK* and *PTEN* levels remained decreased (Figure 6B). Interestingly, unlike the protein levels, *CD31* transcripts were unaffected. *TEK* was therefore chosen for further study. *TEK* levels were upregulated by *KLF4* OE (Figure 6C). Reanalysis of published CHIP-RNA-Seq data from pulmonary arterial endothelial cells (PAEC) with constitutive activation of MEK5 as a KLF4 inducer (24) showed enrichment of KLF4 on active enhancer regions within the *TEK* gene (Figure 6D), suggesting direct transcriptional regulation.

To test if TEK was required for KLF4-dependent Akt activation, we overexpressed *KLF4* in *CTRL* and *TEK* siRNA HUVECs and exposed cells to flow for 4 hours. *KLF4* OE increased TEK protein levels and *TEK* KD strongly inhibited both flow- and *KLF4* OE–induced Akt activation (Figure 6E; quantified in Figure 6F). Thus, *TEK* is a direct KLF4 target that is required for Akt activation. To test these conclusions in vivo, we labeled *fl/fl*, *Smad4*^iΔEC^, and *Klf4*^iΔEC^ retinas for TIE2 protein (Figure 6G). TIE2 was downregulated in retinal ECs (green arrows) but specifically maintained in AVM regions (yellow arrow) (Figure 6G; quantified in Figure 6H), confirming previous findings (20). In *Klf4*^iΔEC^ retinas, TIE2 expression was downregulated (Figure 6G; quantified in Figure 6H). These results were therefore consistent with flow-induced KLF4-dependent upregulation of TIE2 within the AVMs to hyper-activate Akt and amplify EC flow responses.

### Increased EC proliferation-mediated loss of arterial identity drives AVMs.

**Figure 7:**
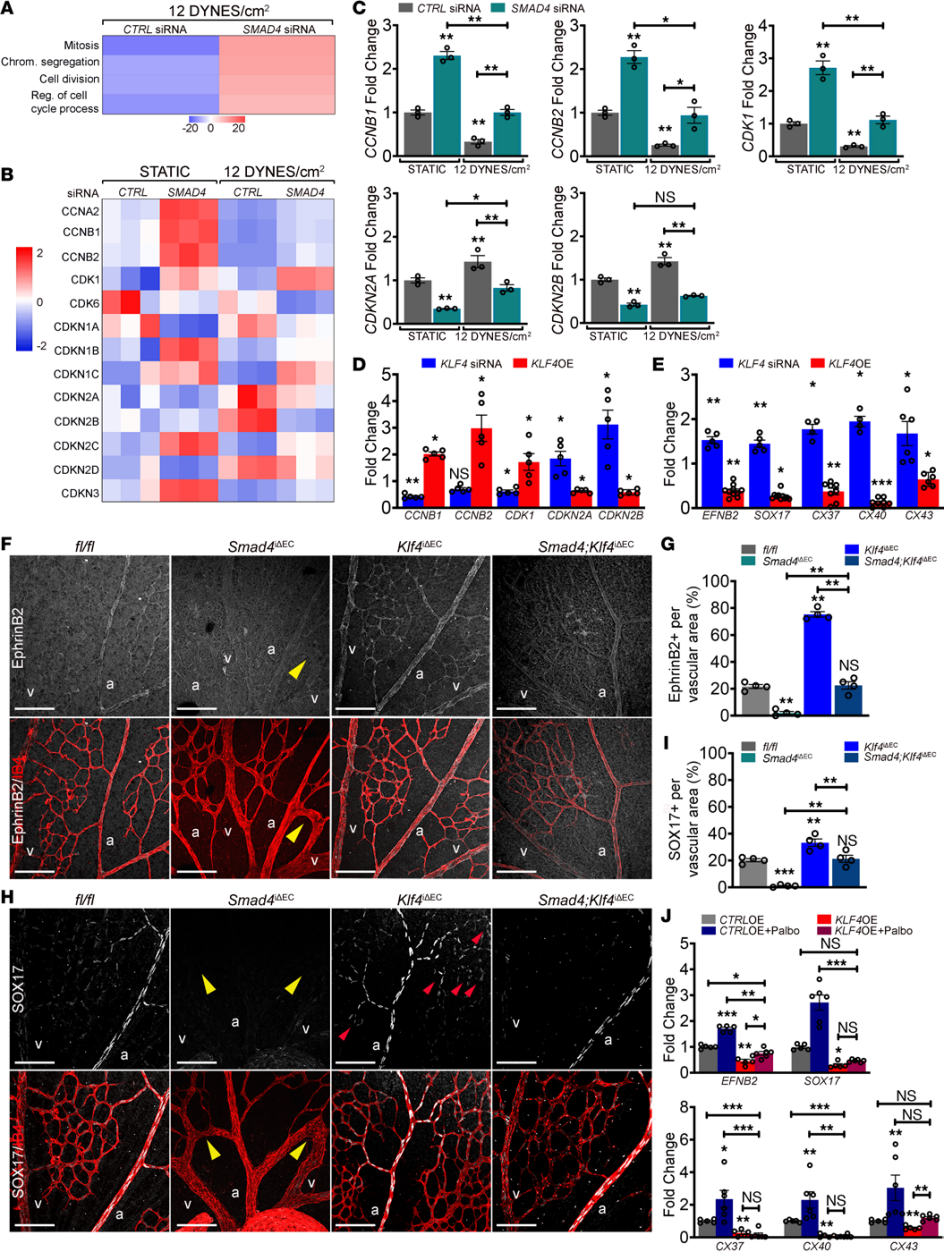
*Smad4* LOF blocks flow-mediated cell cycle arrest resulting in loss of arterial identity. (**A**) Significantly changed GO terms in *CTRL* versus *SMAD4* siRNAs HUVECs subject to 12 DYNES/cm^2^. *n* = 3/group. (**B**) Heatmap for the expression of cell cycle regulators in *CTRL* versus *SMAD4* siRNAs HUVECs grown in static and subject to 12DYNES/cm^2^ (*n* = 3/group). Color key shows log_2_ changes. (**C**) qPCR for *CCNB1, CCNB2, CDK1*, *CDKN2A,* and *CDKN2B* in *CTRL* versus *SMAD4* siRNAs HUVECs grown in static versus subject to 12DYNES/cm^2^ (*n* = 3/group). (**D** and **E**) qPCR for *CCNB1, CCNB2, CDK1*, *CDKN2A,* and *CDKN2B* (**D**) and for *EFNB2*, *SOX17, CX37, CX40,* and *CX43* (**E**) in *KLF4* siRNAs and *KLF4* OE as fold change in relation to *CTRL* siRNA and *CTRL-*OE HUVECs (*n* = 5–10/group). (**F** and **H**) Representative confocal images of labeled retinas for EphrinB2 (white) (**F**), SOX17 (white) (**H**) and IB4 (red) from Tx induced P6 *fl/fl*, *Smad4*^iΔEC^, *Klf4*^iΔEC^, and *Smad4;Klf4*^iΔEC^ mice. (**G** and **I**) Quantification of EphrinB2 (**G**) and SOX17 (**I**) signals in the vascular plexus from the indicated genotypes (*n* = 4 retinas/group). (**J**) qPCR for *EFNB2*, *SOX17, CX37, CX40*, and *CX43* in *KLF4* OE and Palbociclib treated HUVECs (*n* = 5–6/group). Scale Bars: 100μm in **F** and **H**. a, artery; v, vein. Mann Whitney test (**D** and **E**) and 1-way Anova (**C**,**G**,**I**,**J**) were used to determine statistical significance.Data are represented as mean ± SEM. *P < 0.05, **P < 0.01, ***P < 0.001.

Current models propose that decreased polarization and migration of ECs against the direction of flow mediates AVM formation upon *Eng* and *Alk1*EC KOs (17, 21). However, data herein show that *Smad4* depletion increased polarization in flow, arguing that these morphological effects are unlikely to drive AVM development. Remarkably, the gene ontology (GO) terms derived from transcriptomic data for cells under P-FSS with and without *SMAD4* KD suggest a switch from the flow-induced cell cycle arrested state to cell cycle progression (Figure 7A), consistent with results in Figure 1, O–Q and Supplemental Figure 2. We therefore considered the role of EC cell cycle in AVMs.

Cell cycle progression is regulated by cyclins, cyclin-dependent kinases (CDKs) and CDK inhibitors (CKIs). In our RNA-Seq data, *SMAD4* KD blocked the flow-mediated repression of cyclin A2, B1 and B2 (*CCNA2*, *CCNB1*, and *CCNB2*), and the flow-mediated induction of the CKIs, *CDKN1A* (P21), *CDKN2A* (P16), *CDKN2B* (P15), and *CDKN2D* (P19) (heatmap in Figure 7B). These results support the notion that SMAD4 is required for flow-dependent cell cycle arrest in G1. Validation by qPCR confirmed loss of flow-mediated repression of cyclin *CCNB1* and *CCNB2*, of the M phase driver*, CDK1,* and of flow-mediated induction of the CKIs, *CDKN2A* and *CDKN2B* upon *SMAD4* depletion (Figure 7C). *CCNA2*, *CDKN1A*, and *CDKN2D* were unaffected by both parameters (Supplemental Figure 6A).

To identify cell cycle regulators that require KLF4, we assessed expression of these key cell cycle regulators in *KLF4-*KD and *KLF4-*OE cells by qPCR. *CCNB1* and *CCNB2* cyclins and *CDK1* were increased in *KLF4-*OE cells, whereas *CDKN2A* and *CDKN2B* were decreased (Figure 7D), consistent with a role for KLF4 in driving proliferation. These results support a model in which loss of *Smad4* upregulates KLF4 to overcome flow-induced cell cycle arrest; loss of this mechanism thus contributes to AVMs.

Cell cycle arrest in G1 associated with P-FSS also promotes arterial identity (2). Further, the arterial differentiation program requires suppression of endothelial cell-cycle progression and metabolism (30). Conversely, dysregulated BMP9-SMAD4 signaling leads to loss of arterial and gain of venous identity (13, 15, 16). To further untangle the connection between dysregulated cell cycle and arterial-venous specification in this context, we assessed the effect of KLF4 on expression of identity genes by qPCR. The arterial markers *SOX17*, *EFNB2,* Connexin *37 (CX37), CX40,* and *CX43* increased upon *KLF4* KD and decreased upon OE (Figure 7E).

To test these results in vivo, we labeled retinas for EphrinB2 and SOX17 (Figure 7, F and H) and for CX37 and CX40 (Supplemental Figure 6, B and D). In *fl/fl* retinas, all markers were confined to ECs in main arteries and few arterioles. In AVMs, EphrinB2, SOX17, and CX40 expression were abrogated, while downregulated CX37 was still present in arteries engaged in AVMs (Figure 7, F–I and Supplemental Figure 6, B–E). In *Klf4*EC-KO retinas, EphrinB2 was upregulated in all ECs, including veins, and SOX17 expression expanded toward the veins and capillary ECs, whereas CX37 and CX40 were upregulated within the arterial ECs. *Klf4* inactivation in *Smad4*^iΔEC^ retinas largely restored the normal expression patterns of EphrinB2, SOX17, CX37, and CX40 in arteries (Figure 7, F–I and Supplemental Figure 6, B–E).

To further test whether loss of arterial identity is dependent on cell cycle arrest, we treated *KLF4-*OE HUVECs with the CDK inhibitor Palbociclib that blocks cell cycle progression and induces arterial identity (2). We found that the effect of *KLF4* on *SOX17*, *CX37,* and *CX40* was unaffected, whereas Palbociclib reversed the effect of *KLF4* OE on *EFNB2* and *CX43* expression (Figure 7J). Together, these results suggest that increased EC proliferation-mediated loss of arterial identity as a result of excessive KLF4 activation contributes to AVM formation.

**Figure 8:**
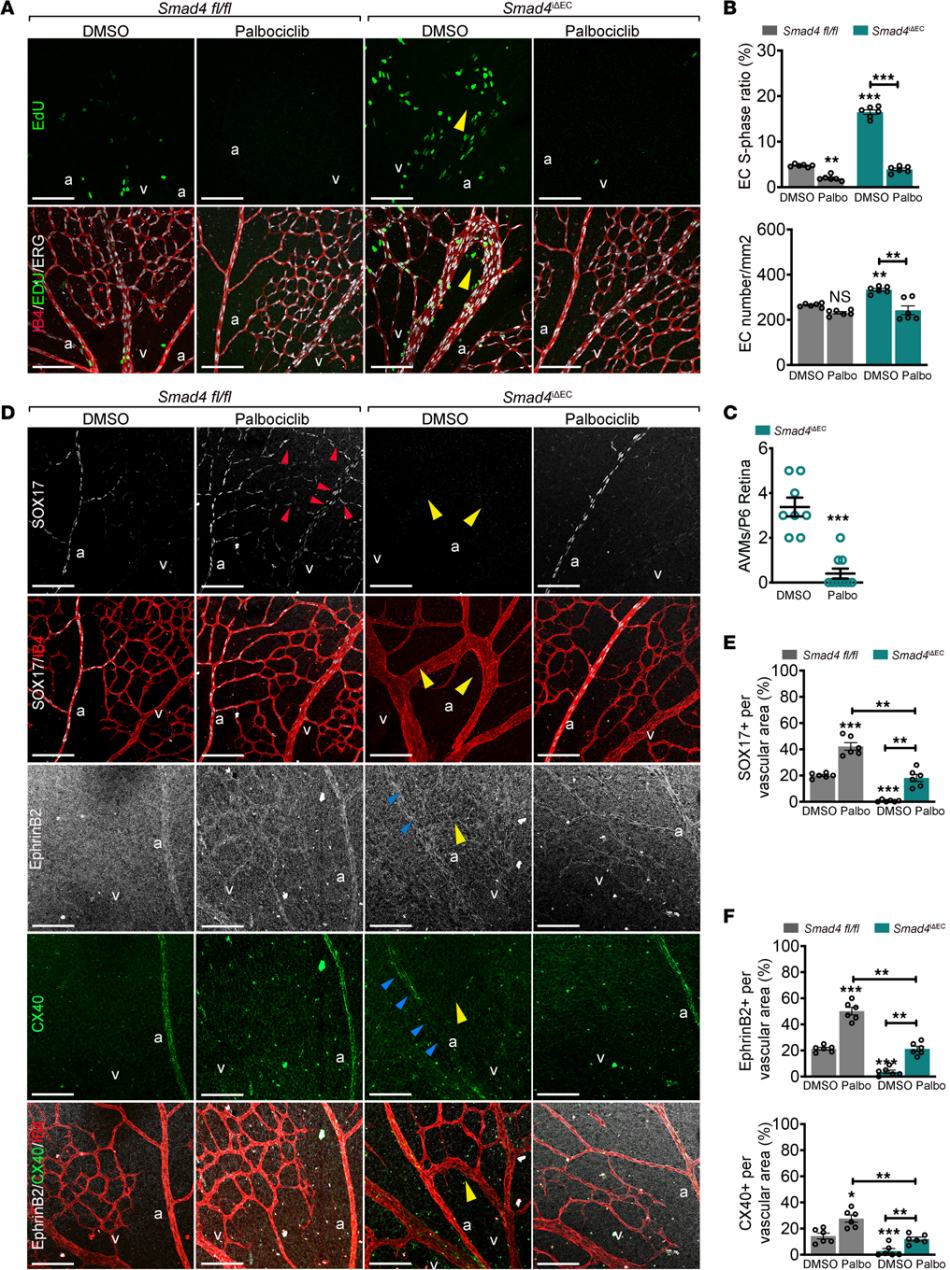
G1 arrest restores arterial identity and rescues AVM formation. (**A**) Confocal images of P6 *Smad4^fl/fl^* and *Smad4*^iΔEC^ retinas treated with DMSO or Palbociclib labeled for EdU (green; upper panel) and EdU (green), ERG (white) and IB4 (red) (lower panel). (**B**) Quantification of the number of EdU^+^/ERG^+^ ECs per total number of ERG^+^ ECs (%) and the total number of ECs. (**C**) Quantification of the number of AVMs in the vascular plexus from indicated genotypes. (**D**) Representative confocal images of retinas stained for SOX17 (white) and IB4 (red), and EphrinB2 (white), CX40 (green), and IB4 (red) from the indicated genotypes, DMSO versus Palbociclib treated. (**E** and **F**) Quantification of SOX17 (**E**), EphrinB2, and CX40 signals (**F**) per vascular area in the plexus of *Smad4*^iΔEC^ retinas DMSO versus Palbociclib treated (n =6 images/3 retina/group). Yellow arrows in **A** and **D** mark AVMs. Red and blue arrows in **D** point to gain (red) and loss (blue) of SOX17, Ephrinb2, and CX40 expression in vascular plexus. Scale Bars: 100μm in **A** and **D**. a, artery; v, vein. Mann-Whitney test (**C**). 1-way Anova (**B**, **E**, and **F**) were used to determine statistical significance. Data are represented as mean ± SEM. *P < 0.05, **P < 0.01, ***P < 0.001.

Lastly, we investigated whether cell cycle arrest, arterial identity, and AVMs are functionally related in vivo by treating *Smad4^fl/fl^* and *Smad4*^iΔEC^ pups with Palbociclib. We first confirmed efficacy of Palbociclib in inducing cell cycle arrest in lungs isolated from treated pups. Palbociclib treatment decreased the expression of CDK4 and CDK6 and inhibited activation of CDK2 (p-CDK2) (Supplemental Figure 7A). In vascular plexus labelled for EdU, ERG, and IB4, Palbociclib decreased the number of EdU^+^ cells in both *fl/fl* and *Smad4*^iΔEC^ retinas and the number of ECs in *Smad4*^iΔEC^ plexus and strongly inhibited AVM formation (Figure 8A; quantified in Figure 8, B and C). In addition, Palbociclib also rescued the excessive vascular front density by blocking EC cycle progression and decreasing the total number of ECs in *fl/fl,* but not in *Smad4*^iΔEC^ retinal front (Supplemental Figure 7, B and C).

Similar to loss of *Klf4,* Palbociclib treatment led to expansion of SOX17 and EphrinB2 expression into capillary and venous ECs in *fl/fl* retinas and upregulation of CX40 in the arteries, as well as restoring their expression in *Smad4*EC-KO arteries (Figure 8D; quantified in Figure 8, E and F). Collectively, these results suggest that loss of arterial identity due to dysregulation of the cell cycle is the central cell event in AVM formation.

## Discussion

It has been proposed that blood flow is ‘a second hit’ in HHT, as murine AVMs develop in regions of high FSS (8, 16), but the mechanisms by which shear stress contributes to AVM pathogenesis remain largely undefined. ECs display an intrinsic FSS set point that determines the signaling and gene expression outputs that control EC phenotype. VEGFR3 expression is one factor that can determine the set point for different types of vessels (6). Also, noncanonical WNT signaling was proposed to modulate axial polarity set point to control vessel regression in low flow regions (7).

We report here that loss of Smad4 triggers a shift in the FSS set point, as *Smad4*-depleted ECs display increased sensitivity to FSS with enhanced elongation and polarization together with FSS-mediated cell cycle blockade at a much lower shear. These results support the concept that ECs within AVMs sense that the flow as above the set point and activate a remodelling program in an attempt to restore FSS to the physiological range. The results also imply that Smad4 signaling is another mechanism that sets the set point to determine flow-mediated EC quiescence responses, e.g., elongation, alignment and orientation, growth suppression, and arterial EC fate. Thus, in *Smad4*-deficient ECs, FSS stimulates abnormal responses typical of higher FSS, consistent with previous work showing that FSS that is well above normal levels induces EC proliferation instead of arrest.

We previously reported that loss of BMP9-SMAD4 signaling leads to PI3K/Akt activation in part through transcriptional derepression of CK2 and resultant inactivation of PTEN (15, 16). Herein, we provide genetic evidence that upregulation of KLF4 drives an increase in TIE2 expression that contributes to PI3K/Akt signaling. These events are specific to AVMs, suggesting that high FSS is also required. These findings thus explain the reported rescue of AVMs by Angiopoietin-2–blocking (Angpt2-blocking) antibodies (20). Interestingly, FSS-induced KLF4 also requires PECAM at cell junctions, consistent with the finding that a complex PECAM-TIE2 is upstream of flow-induced PI3K activation (31). Most likely, the increased activation through TEK and decreased hydrolysis through PTEN inhibition combine to drive Akt activity in AVMs.

Interestingly, identification of excessive KLF4-TIE2-PI3K/Akt signaling in high-flow AVMs implies mechanistic similarities with low-flow cerebral cavernous malformations (CCMs) and venous malformations (VMs). In CCM lesions, malformations are initiated by mutation of *CCM1, 2,* or *3*, but gain-of-function (GOF) mutations in *PI3K* act as a third genetic hit (after clonal loss of the second CCM allele) (32). KLF4 and its close homolog KLF2 are also highly induced in CCMs and contribute to lesion formation (33). VMs also arise as a result of LOF mutations in *PTEN* or GOF mutations in *PI3K* or *TEK* (34).

It is generally assumed that the mechanisms of AVM formation are similar if not identical in HHT1, HHT2, and JP-HHT. Our data, however, show that effects on EC polarization are essentially opposite. Whereas *Smad4* LOF increased elongation, alignment, and polarization in response to FSS, *Eng* and *Alk1* LOF mutations blocked these responses, which is proposed to promote AVM formation (17, 21). Similar cellular defects were suggested to be responsible for increased coronary arteries upon inactivation of embryonic *Smad4* in sinus venosus (35). However, the results presented here argue that elongation and polarization do not drive AVMs; indeed, the notion that they do so is based on correlation without functional or molecular support.

Searching for other mechanisms that may underly AVM development, deregulated proliferation and arterial-venous identity are attractive candidates, as lesions require both increased cell number and direct contact of arterial and venous ECs. Previous work showed loss of arterial identity and gain of venous markers in AVMs (13, 15, 16), which contained exclusively venous-like ECs (5). Additionally, arterial identity is linked to cell cycle arrest (36). P-FSS induces cell cycle arrest in late G1 to maintain arterial identity via a NOTCH-CX37-P27 signaling axis (2), and NOTCH and SMAD1/5 coregulate a number of genes, raising the possibility that these 2 pathways function together (37). Yet, rather than directly inducing Notch-dependent arterial differentiation, recent data show that suppression of EC cycle progression and metabolism are instead required to maintain an arterial differentiation program (30).

**Figure 9:**
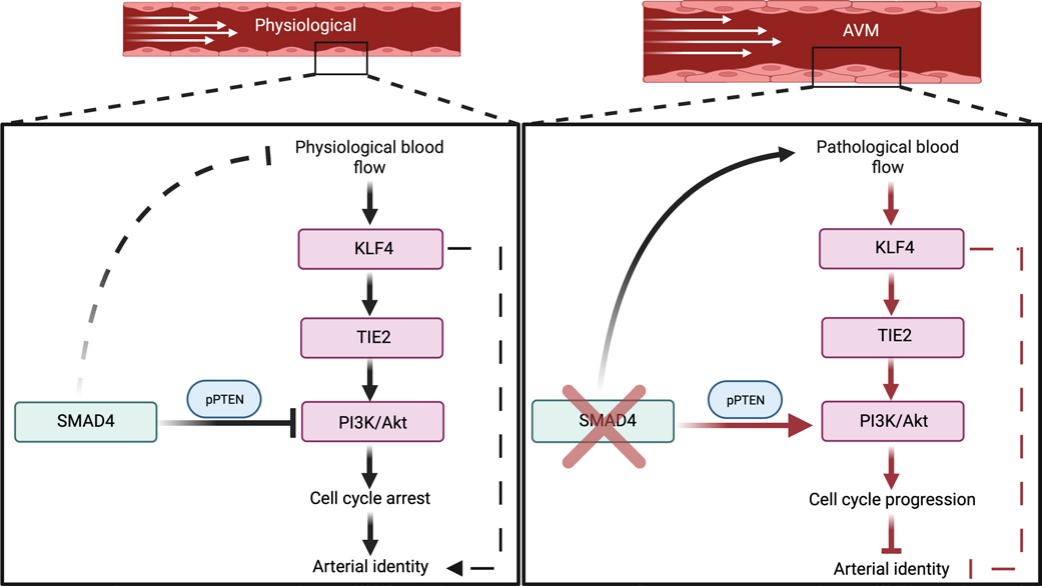
Working model for SMAD4-FSS crosstalk in maintaining EC quiescence: SMAD4 restricts flow-induced KLF4-TIE2-Akt activation to promote EC cycle arrest in G1 and maintenance of arterial identity under P-FSS. Loss of *Smad4* results in overactivation of KLF4-TIE2-Akt signaling in response to pathological shear stress leading to cell cycle progression–mediated loss of arterial identity and AVM formation.

Interestingly, we found that SMAD4 played a critical role in maintaining the P-FSS-induced G1 cell cycle arrest. SMAD4 restricted flow-induced KLF4-TIE2-PI3K/Akt signaling to induce cell cycle arrest by upregulation of the *CDKN2A* and *CDKN2B* CKIs to maintain arterial identity. Loss of *Smad4* led to a switch from P-FSS-mediated inhibition of EC proliferation into stimulation, resulting in AVMs with features of high-flow remodeling. We suggest that chronic, pathological stimulation of this pathway results in loss of arterial identity through elevated KLF4-TIE2-PI3K/Akt (Figure 9). Genetic inactivation of *Klf4* or pharmacological inhibition of PI3K or CDKs thus restored cell cycle arrest in G1 and arterial identity in *Smad4*EC-KO vessels.

It remains possible that loss of flow-driven migration contributes to lesions driven by mutation of *Alk1* or *Eng*, implying distinct mechanisms for AVM formation due to different mutations. But, at present, the hypothesis that migration direction is not the key event triggering AVMs appears both simpler and consistent with older studies demonstrating that, depending on variables such as animal species, age, and specific vascular location, ECs can migrate either against or with the flow (3, 38).

Our findings raise a number of questions. Why does KLF4 induced by physiological flow stabilize vessels while higher levels promote cell proliferation and contribute to pathologies? How do KLF4 and downstream Akt regulate cell cycle progression and arterial identity? Do BMP and NOTCH signaling converge to protect against AVM formation? Further work will be required to elucidate the mechanism upstream of KLF4 by which SMAD4 sets the set-point for FSS-mediated EC responses to maintain EC quiescence.

Although AVMs in patients with HHT form later in life, this is likely due to the requirement for second-hit mutations that yield the homozygous mutant clones that initiate lesions. These pathways are thus likely to be relevant to human disease that afflicts mature vasculature. Targeting the KLF4-TIE2-PI3K/Akt-CDKs axis may be a novel approach for developing new therapeutics for vascular malformations.

## Methods

### RNA-Seq.

For RNA-Seq of HUVECs, RNA was isolated with the Quick-RNA Miniprep Kit (Zymo Research) 60 hours after transfection of *CTRL* or *SMAD4* siRNA grown in static versus 12 DYNES/cm^2^. The RNA 6000 Nano Kit (Agilent) was used to assess the RNA integrity on a Bioanalyzer 2100 (Agilent). Both sequencing and library preparation were performed on the BGISEQ-500 platform.

### RNA-Seq data analysis.

Quality of RNA-Seq reads was assessed with the MultiQC tool (v1.13) and trimmed of adapters using Trimmomatic (v0.39). Reads were mapped by STAR (v2.7.10a) with the following settings: -alignIntronMin 20 and -alignIntronMax 500,000 to the hg38 reference genome. Tag directories were created with makeTagDirectory and reads were counted by the analyzeRepeats.pl function (rna hg38 -strad both -count exons -noadj) both from HOMER (v4.7.2). Differential expression was quantified and normalized using DESeq2. Rpkm.default from EdgeR was used to determine average reads per millions mapped (RPKM). Heatmaps were created by using heatmapper.ca from the RPKM values and represent the row-based Z-scores.

### Animal Experiments.

Deletion of endothelial *Smad4* (*Smad4*^iΔEC^) or *Klf4* (*Klf4*^iΔEC^) was achieved by crossing *Smad4^fl/fl^* (*Smad4^tm2.1Cxd^*; Jackson laboratory) or *Klf4^fl/fl^* (B6.129S6-Klf4^tm1Khk^-Mutant Mouse Resource and Research Center) with Tx-inducible *Cdh5-Cre^ERT2^* mice in a C57bl background. To obtain *Smad4;Klf4*^iΔEC^ double-KO mice, we crossed *Smad4*^iΔEC^ with *Klf4^fl/fl^* mice. Gene deletion was achieved by i.p. injections of 100 μg Tx (Sigma-Aldrich) into *Smad4*^iΔEC^, *Klf4*^iΔEC^, and *Smad4*^iΔEC^*;Klf4*^iΔEC^ at postnatal days (P0–P2). Tx-injected Cre-negative littermates (*fl/fl*) were used as controls. The PI3K inhibitor Pictilisib (Selleckchem; 20 mg/kg/day) and CDK4/6 inhibitor Palbociclib (Selleckchem; 70 mg/kg/day) were administered i.p. at P4 and P5.

### Reagents and antibodies.

For immunodetection, the following antibodies were used: anti-VE-Cadherin (Cell Signaling no. 2500S; 1:600), anti-PECAM (Santa Cruz no. sc-32732; 1:100), Isolectin B4 (IB4; Life Technologies no. 121412; 10 μg/ml), anti-GOLPH4 (Abcam no. ab28049; 1:200), anti-GM130 (BD Bioscience no. 610823; 1:600), anti-ERG (Abcam no. 92513; 1:200), anti-ERG-647 (Abcam no. 196149; 1:100), anti-KLF4 (R&D Systems no. AF3158; 1:200), anti-phospho S6 (pS6; Cell Signaling no. 5364; 1:200), anti-SOX17 (R&D Systems no. AF1924; 1:200), anti-CX37 (Invitrogen no. 40-4200; 1:200), anti-CX40 (Biotrend/Alpha Diagnostics no. CX40-A; 1:200), anti-PH3 (Sigma-Aldrich no. 06-570; 1:200), and anti-Ephrinb2 (R&D Systems no. AF496; 1:100).

For Western blotting, the following antibodies were used: anti-pAkt (Cell Signaling no. 4060; 1:1,000), anti-Akt (Cell Signaling no. 4060; 1:1,000), anti-SMAD4 (Cell Signaling no. 38454S; 1:1000), GAPDH (Sigma-Aldrich no. 5174S; 1:5,000), ACTIN (Sigma-Aldrich no. A1978; 1:1,000), anti-pCDK2 (Cell Signaling no. 2561; 1:1,000), anti-TIE2 (R&D Systems no. AF313; 1:1000), CDK6 (Cell Signaling no. 3136; 1:1,000), and CDK4 (Cell Signaling no. 12790; 1:1,000).

Appropriate secondary antibodies were fluorescently labelled (Alexa Fluor donkey anti-rabbit, Thermo Fisher Scientific no. R37118; Alexa Fluor donkey anti-goat 555, Thermo Fisher Scientific no. A-21432, 1:250) or conjugated to horseradish peroxidase for Western blotting (anti-rabbit no. PI-1000-1, anti-goat no. PI-9500-1, and anti-mouse no. PI-2000-1 IgG (H+L), 1:5,000; all from Vector Laboratories).

### mLECs isolation.

mLECs were isolated from collagenase I–digested lung tissue using rat anti-mouse CD31 monoclonal antibody-coated Dynabeads (11035, Invitrogen) and directly used for RNA analysis.

### Quantitative real-time PCR.

RNAs from HUVECs or mouse lung ECs (mLECs) were purified using RNeasy-kit (Qiagen). The RNA was reverse transcribed High-Capacity cDNA Reverse Transcription Kit (Thermo Fisher Scientific) and qPCR were performed using PowerUP SYBR Green Master Mix (Thermo Fisher Scientific) with a QuantStudio 3 (Thermo Fisher Scientific) according to the manufacture’s protocol. For the detailed list of primer sequences, see Supplemental Table 1.

### Immunostaining.

Eyes of P6 pups were fixed in 4% PFA for 17 minutes at room temperature (rt). After several washes with PBS, dissected retinas were incubated with specific antibodies diluted in blocking buffer (1% FBS, 3% BSA, 0.5% Triton X-100, 0.01% sodium deoxycholate, 0.02% sodium azide in PBS at pH 7.4 [all from Sigma-Aldrich]) at 4°C overnight. The following day, retinas were washed and incubated with IB4 together with the corresponding secondary antibody in PBLEC buffer (1 mM CaCl_2_ [Sigma-Aldrich], 1 mM MgCl_2_ [PanReac AppliChem], 1 mM MnCl_2_ [Sigma-Aldrich] and 0.25% Triton X-100 [Sigma-Aldrich] in PBS) for 1 hour at rt and mounted in fluorescent mounting medium (RotiMount FluorCare; CarlRoth). High-resolution pictures were acquired using Zeiss LSM800 confocal microscope with Airyscan Detector and the Zeiss ZEN software. Quantification of retinal vasculature was done using Fiji.

### Cell culture, siRNA transfection, overexpression HUVECs, and PI3K inhibitor treatment.

HUVECs were isolated from the umbilical cords of newborns (approved by the local ethics committee [2012-388N-MA, 08/11/2018, Medical Faculty Mannheim, Heidelberg University, Germany]). A 3-way valve was inserted into the vein and fixed with a zip tie to wash the vein several times until the effluent buffer was transparent or slightly pink. At that point, the vein was closed with a surgical clamp, filled with a 0.2% Collagenase/Dispase (Sigma-Aldrich) solution and incubated at 37°C for 30–45 minutes. Postincubation, the umbilical cord was emptied into 5 mL of FCS and centrifuged at 300*g* for 5 minutes. The cells were then resuspended in 10 mL of Endothelial Cell Growth Medium MV2 with supplemental mix (PromoCell) and 1% Penicillin/Streptomycin (Sigma-Aldrich), and platted on a 10 cm dish and incubated at 37°C (with 5% CO_2_ and 100% humidity). Cells up to passage 4 were used for experiments. Depletion of *SMAD4*, *KLF4*, *CD31*, *AKT1, VEGFR2,* and *CDH5* was achieved by transfecting 25 pmol of siRNA against *SMAD4* (ON-Targetplus Human SMAD4 siRNA Smart Pool; no. L-003902-00-0005), *KLF4* (siGENOME Human *KLF4* siRNA; no. M-005089-03-0005), *CD31* (5′-GGCCCCAAUACACUUCACA-3′), *AKT1* Stealth siRNA (Thermo Fisher Scientific no. VHS40082), *VEGFR2* (Dharmacon/Horizon no. L-003148-00-005), *CDH5* (Dharmacon/Horizon no. L-003641-00-0005) using Lipofectamine RNAiMax (Invitrogen) in 2% OPTI-MEM. Transfection efficiency was assessed by Western blot and qPCR. Experiments were performed 48–72 hours after transfection and results were compared with siRNA *CTRL* (ON-TARGETplus Non-Targeting Pool D-001810-10-05). Inhibition of PI3K was achieved by using Pictilisib (Selleckchem) in a concentration of 75 nM and inhibition of CDK4/6 by using Palbociclib in concentration of 2 μM. Before experiments, cells were starved for 8–10 hours in 2% FCS.

For Generation of the stable *KLF4-*overexpressing cell line, the *KLF4* overexpression plasmid (TRCN0000492053) was obtained from Sigma (Mission TRC3.0, Sigma-Aldrich). For lentivirus packing, briefly, HEK293T cells were cotransfected with the lentiviral vector and packaging plasmids (pCMV-dR8.91 and pCMV-VSV-G) using X-treme GENE 9 reagent (Sigma-Aldrich). Culture supernatant containing viral particles was collected 36 and 72 hours after transfection and concentrated by centrifugation at 3,000*g* for 60 minutes at 4°C. The pellets were resuspended in 1 mL of PBS and stored at –80°C. For virus infection, HUVECs were transduced with optimal volume of lentiviral virus at 50% confluence in MV2 medium and 8 μg/mL Polybrene (Sigma-Aldrich). After 24 hours, the medium containing viral particles was replaced with fresh medium and after an additional 24 hours, the infected cells were selected with 2 μg/mL puromycin for 48 hours.

### Exposure of ECs to increased shear stress.

HUVECs transfected with siRNAs or OE-HUVECs were plated in a 6-well plate and on an orbital shaker (Rotamax120, Heidolph Instruments) at rpm rates previously determined to generate laminar shear stress of 1, 5, or 12 DYNES/cm^2^. Morphological changes upon flow were confirmed in a μ-Slide VI^0.4^ (Ibidi) using a pump system (Ibidi). RNA-Seq, qPCR, and Western blot experiments were performed using the orbital shaker.

### Western blotting.

HUVECs were washed with PBS and lysed with Laemmli buffer (Bio-Rad). Samples were separated on 10% SDS-PAGE gels and transferred on 0.2μm nitrocellulose membranes (GE Healthcare). Western blots were developed with the Clarity Western ECL Substrate (Bio-Rad) on a Luminescent image Analyzer, Fusion FX (Vilber). Bands intensity was quantified using ImageJ.

### Proliferation assay.

Proliferation analysis was performed using Click-iT EdU Alexa Fluor 488 Imaging kit (Life Technologies). P6 pups were injected with 200 μg EdU (5 mg/mL) and sacrificed 4 hours later. For the in vitro experimentation, 10 μM was added in the culture medium. After fixation, retinas or cells were labelled as per manufacturer’s protocol.

### Statistics.

Results are expressed as the mean ± SEM. Statistical analysis was carried out using a Mann–Whitney U test or 1-way ANOVA followed by Tukey’s for multiple comparisons or Kruskal-Wallis using GraphPad Prism (GraphPad Software). A *P* value of less than 0.05 was considered statistically significant.

### Study approval.

Mice were maintained under standard specific pathogen-free conditions, and animal procedures were approved by the animal welfare commission of the Regierungspräsidium Karlsruhe (Karlsruhe, Germany) and the Romanian animal welfare agency (Cluj-Napoca, Romania).

### Data availability.

RNA-Seq data sets have been deposited at Gene Expression Omnibus (GEO) with accession number GSE232443. Values for all the data points in the graphs can be found in the Supplemental Materials in the Supporting Data Values file.

## Author contributions

KB, YL, and JG conducted experiments and acquired and analyzed data. The order within the first authorship was chosen according to the scientific contribution throughout the manuscript. JC, PG, and IM contributed to the investigation. RO conceptualized the project, was the project administrator, supervised and funded the project, and wrote the first draft of the manuscript. MG, GD, MAS, and RO reviewed and edited the manuscript.

## Supplementary Material

Supplemental data

Supporting data values

## Version 1 07/25/2023

In-Press Preview

## Version 2 09/15/2023

Electronic publication

## References

[1] Baeyens N (2016). Endothelial fluid shear stress sensing in vascular health and disease. J Clin Invest.

[2] Fang JS (2017). Shear-induced Notch-Cx37-p27 axis arrests endothelial cell cycle to enable arterial specification. Nat Commun.

[3] McCue S (2006). Shear stress regulates forward and reverse planar cell polarity of vascular endothelium in vivo and in vitro. Circ Res.

[4] Franco CA (2015). Correction: dynamic endothelial cell rearrangements drive developmental vessel regression. PLoS Biol.

[5] Lee HW (2021). role of venous endothelial cells in developmental and pathologic angiogenesis. Circulation.

[6] Baeyens N (2015). Vascular remodeling is governed by a VEGFR3-dependent fluid shear stress set point. Elife.

[7] Franco CA (2016). Non-canonical Wnt signalling modulates the endothelial shear stress flow sensor in vascular remodelling. Elife.

[8] Baeyens N (2016). Defective fluid shear stress mechanotransduction mediates hereditary hemorrhagic telangiectasia. J Cell Biol.

[9] Johnson DW (1996). Mutations in the activin receptor-like kinase 1 gene in hereditary haemorrhagic telangiectasia type 2. Nat Genet.

[10] McAllister KA (1994). Endoglin, a TGF-beta binding protein of endothelial cells, is the gene for hereditary haemorrhagic telangiectasia type 1. Nat Genet.

[11] Teekakirikul P (2013). Thoracic aortic disease in two patients with juvenile polyposis syndrome and SMAD4 mutations. Am J Med Genet A.

[12] Benn A (2020). BMP-SMAD1/5 signaling regulates retinal vascular development. Biomolecules.

[13] Mahmoud M (2010). Pathogenesis of arteriovenous malformations in the absence of endoglin. Circ Res.

[14] Park SO (2009). Real-time imaging of de novo arteriovenous malformation in a mouse model of hereditary hemorrhagic telangiectasia. J Clin Invest.

[15] Ola R (2016). PI3 kinase inhibition improves vascular malformations in mouse models of hereditary haemorrhagic telangiectasia. Nat Commun.

[16] Ola R (2018). SMAD4 Prevents flow induced arteriovenous malformations by inhibiting casein kinase 2. Circulation.

[17] Park H (2021). defective flow-migration coupling causes arteriovenous malformations in hereditary hemorrhagic telangiectasia. Circulation.

[18] Dimmeler S (1998). Fluid shear stress stimulates phosphorylation of Akt in human endothelial cells: involvement in suppression of apoptosis. Circ Res.

[19] Wang C (2013). Endothelial cell sensing of flow direction. Arterioscler Thromb Vasc Biol.

[20] Crist AM (2019). Angiopoietin-2 inhibition rescues arteriovenous malformation in a smad4 hereditary hemorrhagic telangiectasia mouse model. Circulation.

[21] Jin Y (2017). Endoglin prevents vascular malformation by regulating flow-induced cell migration and specification through VEGFR2 signalling. Nat Cell Biol.

[22] Corti P (2011). Interaction between alk1 and blood flow in the development of arteriovenous malformations. Development.

[23] Hamik A (2007). Kruppel-like factor 4 regulates endothelial inflammation. J Biol Chem.

[24] Moonen JR (2022). KLF4 recruits SWI/SNF to increase chromatin accessibility and reprogram the endothelial enhancer landscape under laminar shear stress. Nat Commun.

[25] Akimoto S (2000). Laminar shear stress inhibits vascular endothelial cell proliferation by inducing cyclin-dependent kinase inhibitor p21(Sdi1/Cip1/Waf1). Circ Res.

[26] Peacock HM (2020). Impaired SMAD1/5 mechanotransduction and Cx37 (Connexin37) expression enable pathological vessel enlargement and shunting. Arterioscler Thromb Vasc Biol.

[27] Coon BG (2015). Intramembrane binding of VE-cadherin to VEGFR2 and VEGFR3 assembles the endothelial mechanosensory complex. J Cell Biol.

[28] Tzima E (2005). A mechanosensory complex that mediates the endothelial cell response to fluid shear stress. Nature.

[29] Morello F (2009). Phosphoinositide 3-kinase signalling in the vascular system. Cardiovasc Res.

[30] Luo W (2021). Arterialization requires the timely suppression of cell growth. Nature.

[31] Tai LK (2005). Flow activates ERK1/2 and endothelial nitric oxide synthase via a pathway involving PECAM1, SHP2, and Tie2. J Biol Chem.

[32] Ren AA (2021). PIK3CA and CCM mutations fuel cavernomas through a cancer-like mechanism. Nature.

[33] Zhou ZN (2016). Cerebral cavernous malformations arise from endothelial gain of MEKK3-KLF2/4 signalling. Nature.

[34] Queisser A (2021). genetic basis and therapies for vascular anomalies. Circ Res.

[35] Poduri A (2017). Endothelial cells respond to the direction of mechanical stimuli through SMAD signaling to regulate coronary artery size. Development.

[36] Chavkin NW (2022). Endothelial cell cycle state determines propensity for arterial-venous fate. Nat Commun.

[37] Larrivee B (2012). ALK1 signaling inhibits angiogenesis by cooperating with the Notch pathway. Dev Cell.

[38] Kiosses WB (1997). Evidence for the migration of rat aortic endothelial cells toward the heart. Arterioscler Thromb Vasc Biol.

