# Supplementary material for: SMAD4 maintains the fluid shear stress set point to protect against arterial-venous malformations
Source: J Clin Invest. 2023 Sep 15;133(18):e168352. doi: 10.1172/JCI168352 (PMC10503796; doi:10.1172/JCI168352)
Supplement: Supplemental data [file jci-133-168352-s092.pdf]

SUPPLEMENTAL MATERIAL FOR

SMAD4 maintains the Fluid Shear Stress set point to protect against Arterial-Venous Malformations

Kuheli Banerjee[1†], Yanzhu Lin[1†], Johannes Gahn[1†], Julio Cordero [2,3], Purnima Gupta[1], Islam Mohamed[1], Mariona Graupera[4], Gergana Dobreva[2,3], Martin A. Schwartz[5], Roxana Ola[1*]

Supplemental Figure Legends

Supplemental Figure 1. *SMAD4*KD augments flow-induced gene expression.

(A) qPCR for the most 10 significant upregulated genes upon 24 hours 12 DYNES/cm^2 FSS in *CTRL* versus *SMAD4* siRNA HUVECs (n = 3/group): *ACKR4* (Atypical Chemokine Receptor 4); *APLNR* (Apelin receptor); *FBLN2* (EGF Containing Fibulin Extracellular Matrix Protein 2); *ITGB4* (Integrin Subunit Beta 4); *ELN* (Elastin); *MRAS* (Muscle RAS Oncogene Homolog); *PLCG2* (Phospholipase C Gamma 2); *KLF4* (Kruppel-Like Factor 4); *SLCO2A1* (Solute Carrier Organic Anion Transporter Family Member 2A1); *TNXB* (Tenascin XB). Data are represented as mean ± SEM: ns- non-significant, **P*<0.05,***P*<0.01,****P*<0.001. One-way Anova.

Supplemental Figure 2. Loss of *SMAD4* leads to dysregulated cell cycle in vitro and in vivo.

(A) Representative immunofluorescence staining of *CTRL* versus *SMAD4* siRNAs HUVECs grown in static versus 1 or 12 DYNES/cm^2 for EdU (green) and DAPI (blue). (**B**) Representative co-labeling of postnatal day 6 (P6) Tx induced *Smad4 fl/fl* and *Smad4*$^{i\Delta EC}$ retinas for EdU+ nuclei (green-upper panel), and EdU (green), ERG (white), and Isolectin B4 (IB4-red) (lower panel). (**C**) Quantification of the total number of ECs (ERG+) in representative images shown in **B**. (**D**) S-phase ratio (EdU+/ERG+) per total ECs (ERG+) in capillaries of *Smad4 fl/fl* and *Smad4*$^{i\Delta EC}$ retinas engaged or not in AVMs. (**E**) Representative co-labeling of postnatal day 6 (P6) Tx induced *Smad4 fl/fl* and *Smad4*$^{i\Delta EC}$ retinas for phospho Histone 3 (PH3-blue) (upper panel) and PH3 (blue), ERG (white), and Isolectin B4 (IB4-red) (lower panel). Yellow arrowheads in **B** and **E** mark ECs+ for EdU and PH3, respectively in capillaries. Red/blue arrowheads in **B** mark the EdU+ ECs in arteries/veins. (**F**) M-phase ratio (PH3+/ERG+) per total ECs (ERG+) in capillaries of *Smad4 fl/fl* and *Smad4*$^{i\Delta EC}$ retinas engaged or not in AVMs. n=9 (3 images (200-600 cells/image)/retina/group). Scale Bars: 100µm in **A**,**B**,**E**. **a**: artery, **v**: vein. Data are represented as mean ± SEM. **P*<0.05,***P*<0.01,****P*<0.001. One-way Anova (**C**,**D**,**F**).

Supplemental Figure 3. KLF4 regulates cell size but not VE Cadherin at cell junctions.

(**A**,**B**) Quantification of cell area in *CTRL*, *SMAD4, KLF4* and *SMAD4;KLF4* siRNAs HUVECs subject to 12 DYNES/cm^2 (**A**) and 1 DYNE/cm^2 (**B**) for 24 hours (n=8 images (70-140 cells/image) per 4 independent experiments/group). (**C**) Representative VE-Cadherin staining (negative images) of *CTRL*, *SMAD4, KLF4* and *SMAD4;KLF4* siRNAs HUVECs subject to 12 DYNES/cm^2 for 24 hours. (**D**) Quantification of VE-Cadherin labeling intensity from experiments in **C** (n=6 images per 3 independent experiments/group). Scale Bars: 100μm. Data are represented as mean ± SEM. **P*<0.05,***P*<0.01,****P*<0.001, ns- non-significant. One-way Anova (**A**,**B**,**D**).

Supplemental Figure 4. Validation of KLF4 antibody in vivo.

(**A**) Representative images of the vascular plexus in retinas from Tx induced P6 *Klf4 fl/fl* and *Klf4*$^{i\Delta EC}$ labelled for KLF4 (green) and IB4 (white). Scale Bars: 50 μm.

Supplemental Figure 5. Increased PI3K signaling mediates EC aberrant responses in *Smad4*ECko.

(**A**) Representative WB for pAkt in HUVECs subject to 12 DYNES/cm^2 treated with PBS or Pictilisib (PI3Ki-75nM) for 4 hours (n=3/group). (**B**) Representative VE-Cadherin staining (negative images) of *CTRL* and *SMAD4* siRNAs HUVECs subject to 24 hours 12 DYNES/cm^2 and treated with PBS, PI3K inhibitor or with *AKT* siRNA. Flow direction: right to left. (**C**) Quantification of the length/width ratio (n=6 average of images (70-140 cells/image) per 3 independent experiments/group) and of EC alignment parallel to flow direction (%) (n=12 average of images (100-240 cells/image) per 3 independent experiments/group). (**D**) Representative images of retinas from P6 *Smad4 fl/fl* and *Smad4*$^{i\Delta EC}$ from pups treated with PBS or PI3K inhibitor labeled for ERG (white), GOLPH4 (red) and IB4 (green)-upper panel and ERG (white), GOLPH4 (red) and IB4 (green line)-lower panel. Yellow arrowheads mark the EC orientation within the AVMs. (**E**) Quantification of EC polarization: against or with flow and neutral (non-oriented) in capillaries and AVMs from P6 retinas of *Smad4 fl/fl* and *Smad4*$^{i\Delta EC}$ pups treated with PBS or PI3Ki (n=3 retinas/group). (**F**) S-phase ratio (EdU+/ERG+) per

total ECs (ERG+) in the vascular plexus of *Smad4*$^{i\Delta EC}$ retinas in PBS versus PI3Ki (Pictilisib) treated pups (n=8 (2 images (200-600 cells/image)/retina/group). (**G**) S-phase ratio (EdU+) per total DAPI+ cells in response to 24 hours 12 DYNES/cm^2 of *CTRL* and *SMAD4* siRNAs HUVECs treated with PBS versus PI3Ki (n=6 (2 images (200-300 cells/image)/experiment/group). (**H**,**I**) *KLF4* mRNA expression by qPCR in HUVECs subject to 12 DYNES/cm^2 and treated with PBS versus Pictilisib (**H**) (n=5/group) and in *CTRL*, *CD31*, *KDR*, and *CDH5* siRNAs HUVECs (**I**) (n=4/group). Scale Bars: 100µm in **D**. **a**: artery, **v**: vein. Data are represented as mean ± SEM. *P<0.05,**P<0.01,***P<0.001, ns- non-significant. One-way Anova (**C**,**E**,**G**), Mann-Whitney test (**F**,**H**,**I**).

Supplemental Figure 6. *Klf4* inactivation restores arterial identity in *Smad4*$^{i\Delta EC}$ retinas

(**A**) qPCR for *CCNA2*, *CDKN1A* and *CDKN2D* in *CTRL* versus *SMAD4* siRNAs HUVECs grown in static versus subject to 12 DYNES/cm^2 (n=3/group). (**B**,**D**) Representative confocal images of labeled retinas for CX37 (white) (**B**), CX40 (green) (**D**) and IB4 (red) from Tx induced P6 *fl/fl*, *Smad4*$^{i\Delta EC}$, *Klf4*$^{i\Delta EC}$ and *Smad4;Klf4*$^{i\Delta EC}$. Yellow arrowheads indicate AVMs. Blue arrowheads indicate expression of arterial markers in arteries and arterioli. (**C**,**E**) Quantification of CX37 (**C**) and CX40 (**E**) signals in the vascular plexus from the indicated genotypes (n = 4 retinas/group). Scale Bars in **B**,**D**: 100µm. **a**: artery, **v**: vein. Data are represented as mean ± SEM. n.s- non-significant, *P<0.05, **P<0.01, ***P<0.001. One-way Anova (**A**,**C**,**E**).

Supplemental Figure 7. Palbociclib reduces vascular density in *Smad4*$^{i\Delta EC}$ retinas.

(**A**) Representative WB images for the indicated proteins of whole lung lysates from pups treated with DMSO and Palbociclib (n = 3/group). (**B**) Confocal images of vascular front of P6 Smad4 *fl/fl* and *Smad4*$^{i\Delta EC}$ retinas treated with DMSO or Palbociclib labeled for IB4 (red) - upper panel, EdU (green) and ERG (white) -middle panel and IB4/ERG/EdU (lower panel) of the vascular front in indicated genotypes. (**C**) Quantification of the vascular density, the number of EdU+/ERG+ ECs per total number of ERG+ ECs (%) and of the total number of ERG+ ECs at the vascular front of *Smad4*$^{i\Delta EC}$ retinas

DMSO or Palbociclib treated. Scale Bars in **B**: 50μm. Data are represented as mean ± SEM. n.s- non-significant, *P<0.05, **P<0.01, ***P<0.001. One-way Anova (**C**).

Supplemental Figure 1

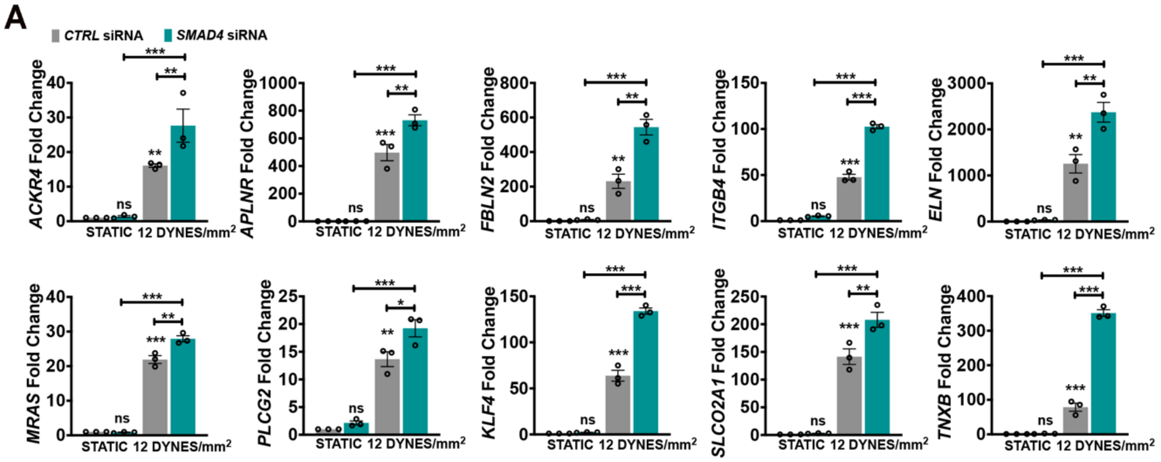

Supplemental Figure 1. *SMAD4*KD augments flow-induced gene expression.
(A) qPCR for the most 10 significant upregulated genes upon 24 hours 12 DYNES/cm^2 FSS in *CTRL* versus *SMAD4* siRNA HUVECs (n = 3/group): *ACKR4* (Atypical Chemokine Receptor 4); *APLNR* (Apelin receptor); *FBLN2* (EGF Containing Fibulin Extracellular Matrix Protein 2); *ITGB4* (Integrin Subunit Beta 4); *ELN* (Elastin); *MRAS* (Muscle RAS Oncogene Homolog); *PLCG2* (Phospholipase C Gamma 2); *KLF4* (Kruppel-Like Factor 4); *SLCO2A1* (Solute Carrier Organic Anion Transporter Family Member 2A1); *TNXB* (Tenascin XB). Data are represented as mean ± SEM: ns- non-significant, **P*<0.05,***P*<0.01,****P*<0.001. One-way Anova.

Supplemental Figure 2

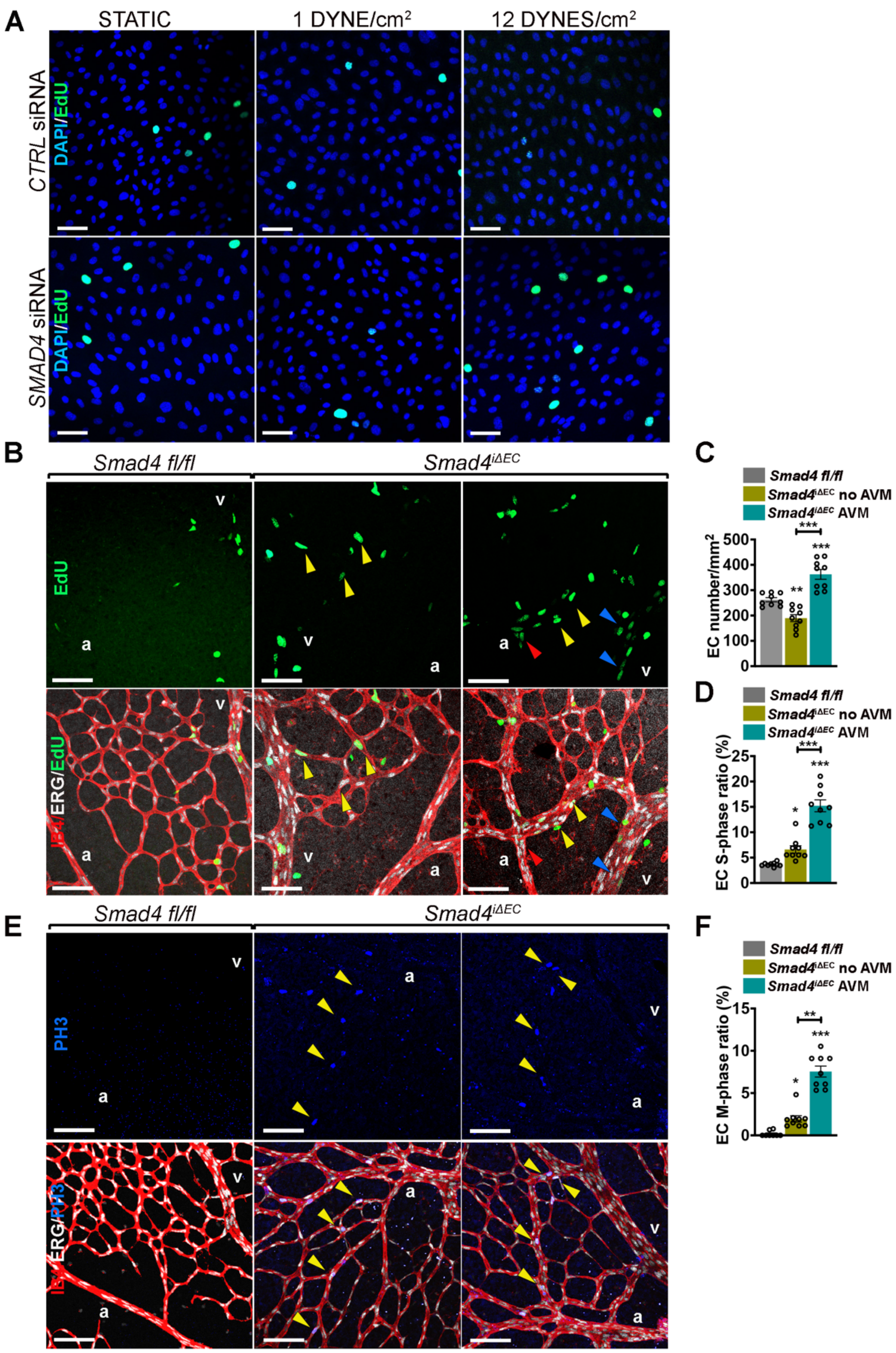

Supplemental Figure 2. Loss of *SMAD4* leads to dysregulated cell cycle in vitro and in vivo. **(A)** Representative immunofluorescence staining of *CTRL* versus *SMAD4* siRNAs HUVECs grown in static versus 1 or 12 DYNES/cm^2 for EdU (green) and DAPI (blue). (**B**) Representative co-labeling of postnatal day 6 (P6) Tx induced *Smad4 fl/fl* and *Smad4*$^{i\Delta EC}$ retinas for EdU+ nuclei (green-upper panel), and EdU (green), ERG (white), and Isolectin B4 (IB4-red) (lower panel). (**C**) Quantification

of the total number of ECs (ERG+) in representative images shown in **B**. (**D**) S-phase ratio (EdU+/ERG+) per total ECs (ERG+) in capillaries of *Smad4 fl/fl* and *Smad4*$^{i\Delta EC}$ retinas engaged or not in AVMs. (**E**) Representative co-labeling of postnatal day 6 (P6) Tx induced *Smad4 fl/fl* and *Smad4*$^{i\Delta EC}$ retinas for phospho Histone 3 (PH3-blue) (upper panel) and PH3 (blue), ERG (white), and Isolectin B4 (IB4-red) (lower panel). Yellow arrowheads in **B** and **E** mark ECs+ for EdU and PH3, respectively in capillaries. Red/blue arrowheads in **B** mark the EdU+ ECs in arteries/veins. (**F**) M-phase ratio (PH3+/ERG+) per total ECs (ERG+) in capillaries of *Smad4 fl/fl* and *Smad4*$^{i\Delta EC}$ retinas engaged or not in AVMs. n=9 (3 images (200-600 cells/image)/retina/group). Scale Bars: 100μm in **A**,**B**,**E**. **a**: artery, **v**: vein. Data are represented as mean ± SEM. *$P<0.05$,**$P<0.01$,***$P<0.001$. One-way Anova (**C**,**D**,**F**).

Supplemental Figure 3

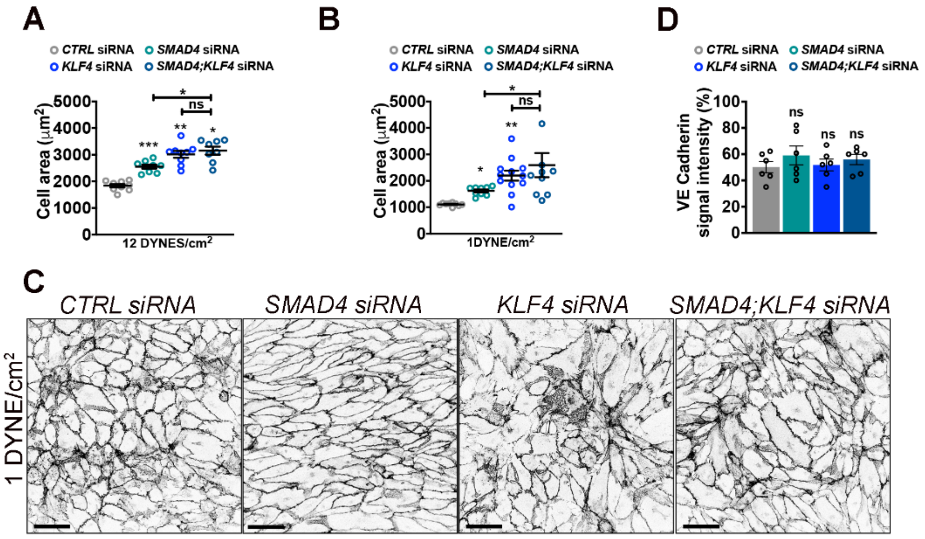

Supplemental Figure 3. KLF4 regulates cell size but not VE Cadherin at cell junctions.
(**A**,**B**) Quantification of cell area in *CTRL*, *SMAD4, KLF4* and *SMAD4;KLF4* siRNAs HUVECs subject to 12 DYNES/cm^2 (**A**) and 1 DYNE/cm^2 (**B**) for 24 hours (n=8 images (70-140 cells/image) per 4 independent experiments/group). (**C**) Representative VE-Cadherin staining (negative images) of *CTRL*, *SMAD4, KLF4* and *SMAD4;KLF4* siRNAs HUVECs subject to 12 DYNES/cm^2 for 24 hours. (**D**) Quantification of VE-Cadherin labeling intensity from experiments in **C** (n=6 images per 3 independent experiments/group). Scale Bars: 100µm. Data are represented as mean ± SEM. **P*<0.05,***P*<0.01,****P*<0.001, ns- non-significant. One-way Anova (**A**,**B**,**D**).

Supplemental Figure 4

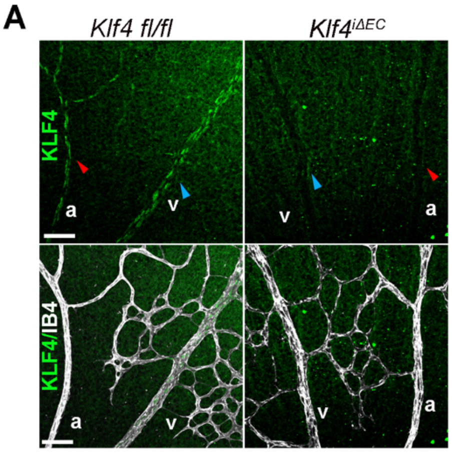

Supplemental Figure 4. Validation of KLF4 antibody in vivo.
(**A**) Representative images of the vascular plexus in retinas from Tx induced P6 *Klf4 fl/fl* and *Klf4*$^{i\Delta EC}$ labelled for KLF4 (green) and IB4 (white). Scale Bars: 50 µm.

Supplemental Figure 5

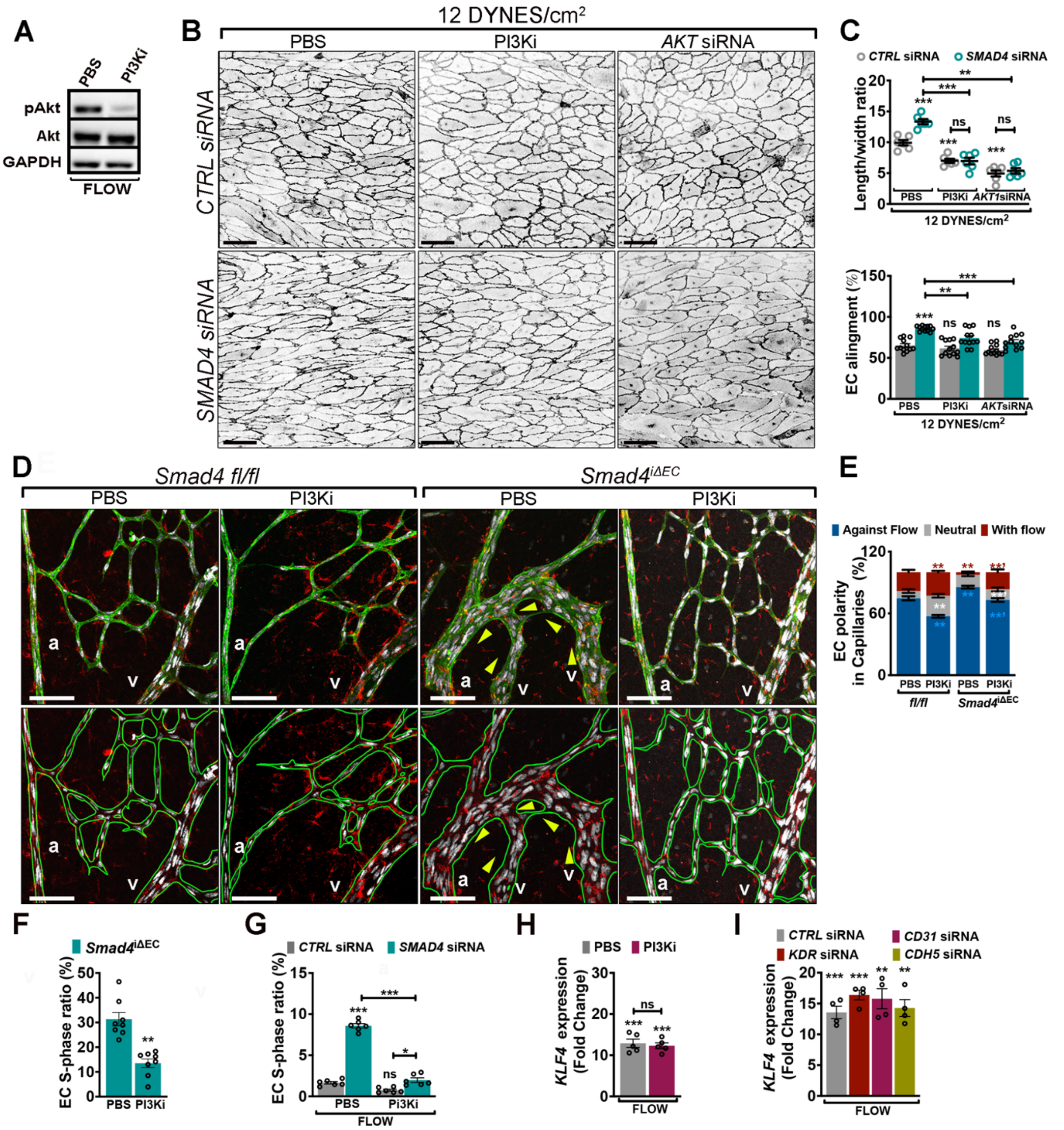

Supplemental Figure 5. Increased PI3K signaling mediates EC aberrant responses in *Smad4*ECko.

(**A**) Representative WB for pAkt in HUVECs subject to 12 DYNES/cm^2 treated with PBS or Pictilisib (PI3Ki-75nM) for 4 hours (n=3/group). (**B**) Representative VE-Cadherin staining (negative images) of *CTRL* and *SMAD4* siRNAs HUVECs subject to 24 hours 12 DYNES/cm^2 and treated with PBS, PI3K inhibitor or with *AKT* siRNA. Flow direction: right to left. (**C**) Quantification of the length/width ratio (n=6 average of images (70-140 cells/image) per 3 independent experiments/group) and of EC alignment parallel to flow direction (%) (n=12 average of images (100-240 cells/image) per 3 independent experiments/group). (**D**) Representative images of retinas from P6 *Smad4 fl/fl* and *Smad4*$^{i\Delta EC}$ from pups treated with PBS or PI3K inhibitor labeled for ERG (white), GOLPH4 (red) and IB4 (green)-upper panel and ERG (white), GOLPH4 (red) and IB4 (green line)-lower panel. Yellow arrowheads mark the EC orientation within the AVMs. (**E**) Quantification of EC polarization: against or with flow and neutral (non-oriented) in capillaries and AVMs from P6 retinas of *Smad4 fl/fl* and *Smad4*$^{i\Delta EC}$ pups treated with PBS or PI3Ki (n=3 retinas/group). (**F**) S-phase ratio (EdU+/ERG+) per total ECs (ERG+) in the vascular plexus of *Smad4*$^{i\Delta EC}$ retinas in PBS versus PI3Ki (Pictilisib) treated

pups (n=8 (2 images (200-600 cells/image)/retina/group). (**G**) S-phase ratio (EdU+) per total DAPI+ cells in response to 24 hours 12 DYNES/cm^2 of *CTRL* and *SMAD4* siRNAs HUVECs treated with PBS versus PI3Ki (n=6 (2 images (200-300 cells/image)/experiment/group). (**H**,**I**) *KLF4* mRNA expression by qPCR in HUVECs subject to 12 DYNES/cm^2 and treated with PBS versus Pictilisib (**H**) (n=5/group) and in *CTRL*, *CD31*, *KDR*, and *CDH5* siRNAs HUVECs (**I**) (n=4/group). Scale Bars: 100µm in **D**. **a**: artery, **v**: vein. Data are represented as mean ± SEM. **P*<0.05,***P*<0.01,****P*<0.001, ns- non-significant. One-way Anova (**C**,**E**,**G**), Mann-Whitney test (**F**,**H**,**I**).

Supplemental Figure 6

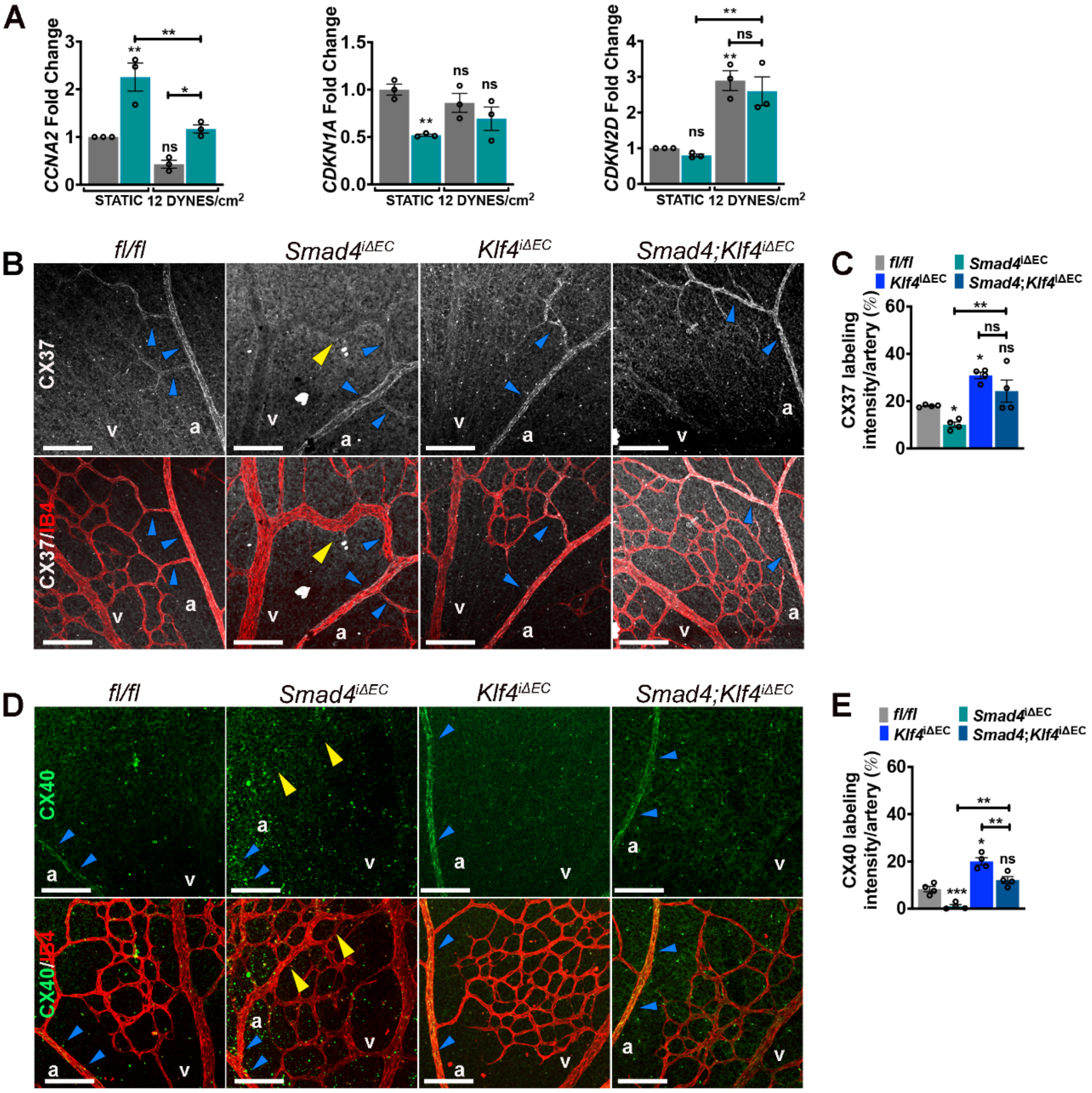

Supplemental Figure 6. *Klf4* inactivation restores arterial identity in *Smad4*$^{i\Delta EC}$ retinas
(**A**) qPCR for *CCNA2*, *CDKN1A* and *CDKN2D* in *CTRL* versus *SMAD4* siRNAs HUVECs grown in static versus subject to 12 DYNES/cm^2 (n=3/group). (**B**,**D**) Representative confocal images of labeled retinas for CX37 (white) (**B**), CX40 (green) (**D**) and IB4 (red) from Tx induced P6 *fl/fl*, *Smad4*$^{i\Delta EC}$, *Klf4*$^{i\Delta EC}$ and *Smad4;Klf4*$^{i\Delta EC}$. Yellow arrowheads indicate AVMs. Blue arrowheads indicate expression of arterial markers in arteries and arterioli. (**C**,**E**) Quantification of CX37 (**C**) and CX40 (**E**) signals in the vascular plexus from the indicated genotypes (n = 4 retinas/group). Scale Bars in **B**,**D**: 100µm. **a**: artery, **v**: vein. Data are represented as mean ± SEM. n.s- non-significant, *$P<0.05$, **$P<0.01$, ***$P<0.001$. One-way Anova (**A**,**C**,**E**).

Supplemental Figure 7

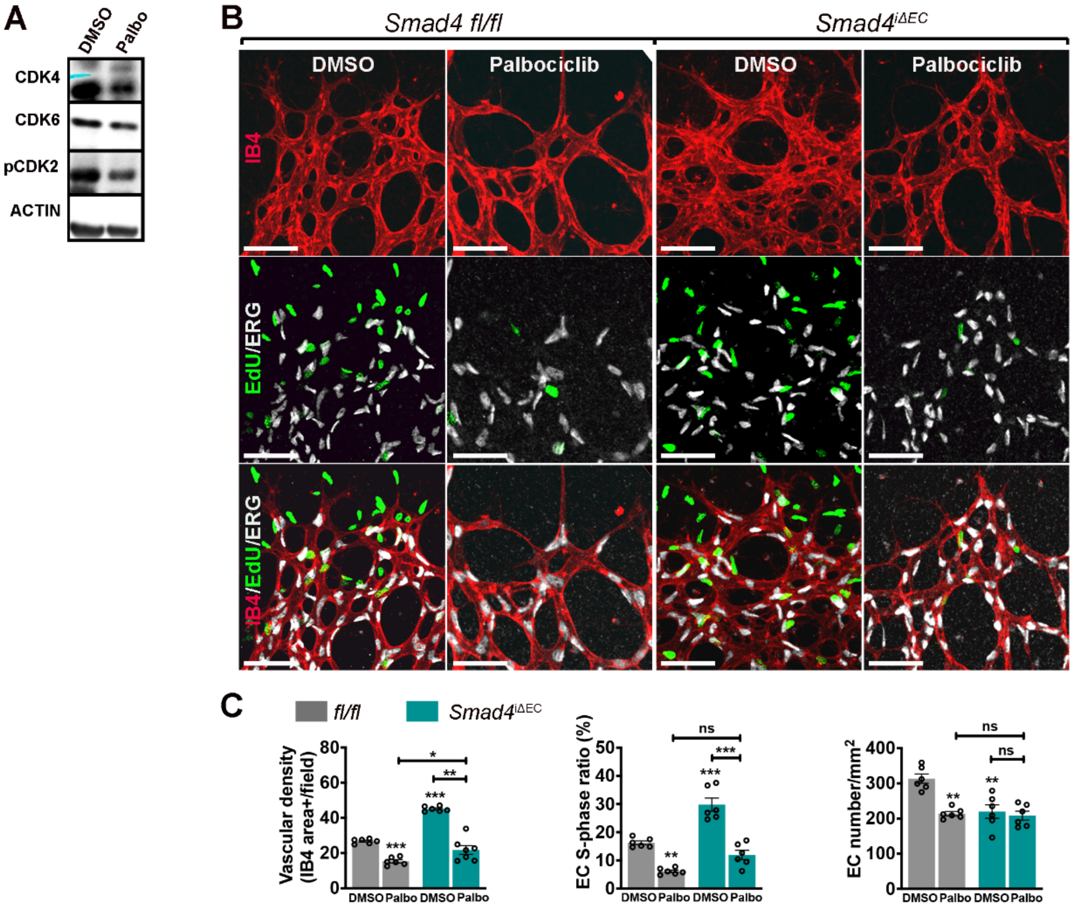

Supplemental Figure 7. Palbociclib reduces vascular density in *Smad4*$^{i\Delta EC}$ retinas.
(**A**) Representative WB images for the indicated proteins of whole lung lysates from pups treated with DMSO and Palbociclib (n = 3/group). (**B**) Confocal images of vascular front of P6 Smad4 *fl/fl* and *Smad4*$^{i\Delta EC}$ retinas treated with DMSO or Palbociclib labeled for IB4 (red) - upper panel, EdU (green) and ERG (white) -middle panel and IB4/ERG/EdU (lower panel) of the vascular front in indicated genotypes. (**C**) Quantification of the vascular density, the number of EdU+/ERG+ ECs per total number of ERG+ ECs (%) and of the total number of ERG+ ECs at the vascular front of *Smad4*$^{i\Delta EC}$ retinas DMSO or Palbociclib treated. Scale Bars in **B**: 50µm. Data are represented as mean ± SEM. n.s- non-significant, **P*<0.05, ***P*<0.01, ****P*<0.001. One-way Anova (**C**).

Supplemental Methods

Supplemental Table1: List of mouse and human primers used in the study.

**Gene name (Protein name)**	**Forward Sequence**	**Reverse Sequence**
**Primers for HUVECs**		
ACKR4 (human-Atypical Chemokine Receptor 4)	GTTTTCGTCATTGGACTTGCAG	GCTACAGCCAAATTCAGGATGT
APLNR (human-Apelin Receptor)	CTCTGGACCGTGTTTCGGAG	GGTACGTGTAGGTAGCCCACA
CCNA2 (human-Cyclin A2)	ACCCAGAAAACCATTGGTCC	CATTTAACCTCCATTTCCCTAAGGT
CCNB1 (human-Cyclin B1)	AATAAGGCGAAGATCAACATG GC	TTTGTTACCAATGTCCCCAAGAG
CCNB2 (human-Cyclin B2)	CCGACGGTGTCCAGTGATTT	TGTTGTTTTGGTGGGTTGAACT
CDK1 (human-CDK1)	AAACTACAGGTCAAGTGGTAGC C	TCCTGCATAAGCACATCCTGA
CDKN1A (human-p21)	TGTCCGTCAGAACCCATGC	AAAGTCGAAGTTCCATCGCTC
CDKN2A (human-p16)	CAACGCACCGAATAGTTACG	AGCACCACCAGCGTGTC
CDKN2B (human-p15)	CACCGTTGGCCGTAAACTTAAC	TAATGAAGCTGAGCCCAGTCT
GJA4 (human- CX37)	ACACCCACCCTGGTCTACC	CACTGGCGACATAGGTGCC
GJA5 (human-CX40)	CCGTGGTAGGCAAGGTCTG	ATCACACCGGAAATCAGCCTG
GJA1 (human-CX43)	GGTGACTGGAGCGCCTTAG	GCGCACATGAGAGATTGGGA
ELN (human-Elastin)	GCAGGAGTTAAGCCCAAGG	TGTAGGGCAGTCCATAGCCA
EPHRINB2 (human-Ephrin B2)	TATGCAGAACTGCGATTTCCAA	TGGGTATAGTACCAGTCCTTGTC
FBLN2 (human-Fibulin 2)	ACTGTGGGTTCTTACCACTGT	CCACCTGGGAAAATTCTGACTT
FLT4 (human-VEGFR3)	TGCACGAGGTACATGCCAAC	GCTGCTCAAAGTCTCTCACGAA
GAPDH (human-GAPDH)	CTGGGCTACACTGAGCACC	AAGTGGTCGTTGAGGGCAATG
HPRT (human-Hypoxanthine Phosphoribosyltransferase 1)	GACCAGTCAACAGGGGACAT	CCTGACCAAGGAAAGCAAAG
ITGB4 (human-Integrin beta-4)	CTCCACCGAGTCAGCCTTC	CGGGTAGTCCTGTGTCCTGTA
KLF4 (human-KLF4)	CCCACATGAAGCGACTTCCC	CAGGTCCAGGAGATCGTTGAA

MRAS (human-Ras-related protein M-Ras)	TTCCTCATCGTCTACTCCGTC	AGGATCATCGGGAATGACTCC
PECAM-1 (human-PECAM1)	AAGTGGAGTCCAGCCGCATATC	ATGGAGCAGGACAGGTTCAGTC
PLCG2 (human-PLCG2)	CATCCTATATGGCACTCAGTTCG	TCCTGGTGTAAGATTTTCAAGCC
SLCO2A1 (human-SLCO2A1)	TCGGTCTTCGGCAACATTAAG	GCTCTTGAAGTAGGCGCTGTA
SOX17 (human-SOX17)	GTGGAACCGCACGGAATTTG	GGAGATTCACACCGGAGTTCA
TEK (human-TIE2)	TTAGCCAGCTTAGTTCTCTGTGG	AGCATCAGATACAAGAGGTAGGG
TNXB (human-TN-X)	GCCCTGCTCACTTGGACTG	GGAGCCGTGCATTGTAGGAG
CDH5 (human-VE-Cadherin)	QT00013244, Qiagen	
KDR (human-VEGFR2)	QT00069818, Qiagen	
SMAD4 (human-SMAD4)	QT00013174, Qiagen	
**Primers for mouse lung ECs**		
Gapdh (mouse-GAPDH)	AGGTCGGTGTGAACGGATTTG	TGTAGACCATGTAGTTGAGGTCA
Klf4 (mouse-KLF4)	QT00095431, Qiagen	
Smad4 (mouse-SMAD4)	QT00130585, Qiagen	
